# Chinese herbal medicine combined with western medicine for the treatment of type 2 diabetes mellitus with hyperuricemia: A systematic review and meta-analysis

**DOI:** 10.3389/fphar.2023.1102513

**Published:** 2023-01-24

**Authors:** Hongyan Liu, Sihan Peng, Haipo Yuan, Yuchi He, Jiao Tang, Xiyu Zhang

**Affiliations:** ^1^ Hospital of Chengdu University of Traditional Chinese Medicine, Chengdu, China; ^2^ TCM Regulating Metabolic Diseases Key Laboratory of Sichuan Province, Hospital of Chengdu University of Traditional Chinese Medicine, Chengdu, China; ^3^ Chengdu University of Traditional Chinese Medicine, Chengdu, China

**Keywords:** type 2 diabetes mellitus, hyperuricemia, Chinese herbal medicine, meta–analysis, systematic review

## Abstract

**Background:** Chinese herbal medicine (CHM) has the advantage of being safe and effective and has been widely used in clinical practice for the treatment of type 2 diabetes mellitus (T2DM) with hyperuricemia (HUA), but its overall efficacy and safety remain unclear. This study aimed to evaluate the efficacy and safety of CHM for the treatment of T2DM with HUA based on randomized controlled trials (RCTs) to provide clinical evidence.

**Methods:** The protocol evaluated in this study is registered with PROSPERO (CRD42022351519). As of November 2022, eight databases were searched, and RCTs of CHM for the treatment of T2DM with HUA were included. Outcome indicators observed included fasting blood glucose (FBG), 2-h postprandial glucose (2hPG), glycated hemoglobin (HbA1c), uric acid (UA), triglycerides (TG), total cholesterol (TC), overall effectiveness, and adverse events. Utilizing Review Manager 5.4, Stata V14.0, and GRADEpro, the included studies were evaluated, and the quality of the evidence was determined.

**Results:** 18 RCTs covering 1,311 patients were included in this study. The results of the study demonstrated that the combination of CHM and western medicine (WM) was more effective in treating patients with T2DM with HUA than WM alone, with significant improvements in FBG (weighted mean differences (WMD) = −0.60.95% confidence interval (CI) [−0.81, −0.40], *p* < 0.00001), 2hPG (WMD = −1.12.95% CI [−1.64, −0.60], *p* < 0.0001), HbA1c (WMD = −0.80.95% CI [−1.04, −0.56], *p* < 0.00001), UA (WMD = −53.47.95% CI [−67.45, −39.48], *p* < 0.00001), TG (WMD = −0.56.95% CI [−0.74, −0.38], *p* < 0.00001), TC (WMD = −0.49.95% CI [−0.65, −0.33], *p* < 0.00001), and overall effective rate (risk ratio (RR) = 1.29.95%CI [1.13, 1.48], *p* = 0.0002). The quality of evidence for all outcomes was low.

**Conclusion:** Compared with WM alone, the combination of CHM and WM was more effective in treating patients with T2DM with HUA, with significant improvements in glucose metabolism, uric acid, and lipids. However, further evaluation by high−quality RCT results is needed due to the low quality and high heterogeneity of the evidence.

**Systematic Review Registration**: [https://systematicreview.gov/], identifier [CRD42022351519].

## 1 Introduction

Type 2 diabetes mellitus (T2DM), a chronic systemic disease characterized by metabolic disorders (Xu et al., 2018), is one of the most challenging global public health issues of the 21st century (Lou et al., 2020). Hyperuricemia (HUA) is a metabolic disorder characterized by abnormally high levels of uric acid in the blood. It is associated with arthritis and the formation of gout stones, with gout further developing HUA (Gois and Souza, 2017; Kojima et al., 2019). The prevalence of HUA in people diagnosed with T2DM ranges from 16% to 38% (Donkeng et al., 2021). There are positive correlation and bidirectional causality between T2DM and HUA (Krishnan et al., 2013; Ahmadieh and Azar, 2017). On the one hand, diabetic insulin resistance competitively inhibits uric acid secretion (Gao et al., 2019). At the same time, hyperinsulinemia to insulin resistance secondary increases uric acid reabsorption and promotes the development of HUA (Li and Ye, 2017). On the other hand, excessive uric acid levels can trigger inflammatory response and oxidative stress that result in insulin resistance (Su et al., 2021); while uric acid leads to pancreatic β−cell dysfunction by decreasing nitric oxide bioavailability (Ghasemi, 2021), two alterations that are central to the physiopathology of T2DM. Another study revealed that the co−existence of T2DM and HUA increased the risk of cardiovascular disease and kidney disease (Viazzi et al., 2017; Lu et al., 2020; Liu et al., 2021), two complications that are significant causes of death and increase the public health burden on society (Xu et al., 2013).

The main risk factors for T2DM and HUA are insulin resistance and obesity, which often co−exist and interact with each other (Li et al., 2013). Due to the increased prevalence and co−existence of T2DM and HUA leading to serious complications and few clinical trials investigating the use of drugs in both diseases (Mortada, 2017), active and effective measures should be sought to improve T2DM combined with HUA. The current treatment of T2DM with HUA with western medicine (WM) is controversial and is associated with unavoidable adverse effects. Available clinical trials have shown that febuxostat and benzbromarone both have uric acid-lowering and hypoglycemic effects (Shang et al., 2017), but the common adverse effects of febuxostat are abnormal liver function, rash, nausea, arthralgia, and high cardiovascular risk (Love et al., 2010; White et al., 2018), while benzbromarone has serious hepatotoxicity and was withdrawn from the European market in 2003 (Azevedo et al., 2014). New glucose−lowering drugs can achieve glucose−lowering and uric acid−lowering effects. However, the current research is small, the applicable population is not limited, and urinary adverse effects may occur (Zhang J. Y. et al., 2022). Some patients may not respond to existing uric acid−lowering drugs (Major et al., 2018), and existing uric acid−lowering drugs have been found to increase cardiovascular events and cause severe liver damage (Zhang J. Y. et al., 2022). The use of uric acid−lowering therapy can increase the risk of hypersensitivity reactions and severe skin reactions if there are comorbidities, predominantly renal and cardiovascular disease (Bardin and Richette, 2017). Uric acid can be obtained through the external intake of purine−rich foods such as seafood and meat, so healthy dietary management is also essential for preventing and treating HUA (Adachi et al., 2017). T2DM and HUA have the potential for a bi−directional causal relationship. They are physiopathologically mutually reinforcing, but there are many constraints on treatment options, so the search for alternative therapies is urgent.

Traditional Chinese medicine (TCM) has been widely used as a complementary and alternative therapy throughout Chinese history. TCM is used in accordance with TCM theory, which obtains clinical information through the four diagnoses and then provides individualized treatment plans for patients. As TCM continues to develop, its clinical value is becoming more recognized worldwide. Chinese herbal medicine (CHM), as the primary therapy of TCM, has the characteristics of being easily accessible, safe, and effective. It has been proved that CHM can exert the efficacy of lowering glucose and lipid, and uric acid through multi−target and multi−channel (Zhou et al., 2013; Li Z. N. et al., 2020). However, there is no systematic evaluation or meta−analysis to date on the efficacy and safety of CHM for the treatment of T2DM with HUA. Therefore, this study aims to evaluate the efficacy and safety of CHM in the treatment of T2DM with HUA and to provide high−quality clinical evidence and scientifically optimized treatment strategies.

## 2 Methods

This systematic evaluation and meta−analysis were performed according to the Preferred Reporting Items for Systematic Evaluation and Meta−Analysis (PRISMA−P) guidelines (Moher et al., 2015) (Supplementary File S1). Moreover, the protocol of this evaluation is registered with PROSPERO (CRD42022351519).

### 2.1 Search strategy

A comprehensive computer search of eight databases was conducted: Pubmed, Cochrane Library, Embase, Web of Science, China National Knowledge Infrastructure (CNKI), Wanfang database, China Biomedical Database (CBM), and China Science and Technology Journal Database (VIP). Ongoing or completed but unpublished randomized controlled trials (RCTs) were also searched on the China Clinical Trials Registry (CHiCTR) (http://www.chictr.org.cn/index.aspx) and ClinicalTrials.gov. The search period is from the establishment of the database until November 2022. All RCTs of CHM for the treatment of T2DM with HUA were included, with no restrictions on language. The search was conducted using a combination of subject terms and free words, including “type 2 diabetes”, “diabetes, type 2″, “hyperuricemia,” “Chinese medicine,” “traditional medicine,” “Chinese herbal medicine,” *etc.* Comprehensive search strategies for the databases are shown in the (Supplementary Table S1).

### 2.2 Inclusion and exclusion criteria

RCTs assessing the effect of CHM on T2DM with HUA were included in this study. The inclusion criteria were as follows: 1) Study format: RCTs; 2) Study subjects: patients with clinically confirmed T2DM with HUA or with gout (regardless of age, gender, race or nationality); 3) Interventions: CHM (regardless of dosage form and duration) combined with WM in the intervention group and WM in the control group; 4) Outcome evaluation indicators: the primary indicators of the study were fasting blood glucose (FBG), 2-h postprandial glucose (2hPG), glycated hemoglobin (HbA1c), and uric acid (UA). The secondary outcome indicators were triglycerides (TG), total cholesterol (TC), overall effective rate and adverse effects. The exclusion criteria were as follows: 1) non−RCTs, such as famous doctors’ experiences, animal experiments, retrospective studies, reviews, and other types of studies; 2) repeated publications; 3) lack of article results or data; and 4) Other TCM interventions besides CHM, such as acupuncture, moxibustion, and tui na, as well as herbal enemas.

### 2.3 Literature screening and data extraction

Two investigators (SP and YH) screened the literature by pre−established inclusion and exclusion criteria and imported the literature to be screened into EndNote X9 software to manage the literature. Two reviewers (HY and JT) independently extracted relevant data to the data statistics table, which included: the first author, year of publication, sample size, patient characteristics (age, gender, duration of disease, *etc.*), interventions (drug name, composition, *etc.*), duration of treatment, outcome indicators, and adverse effects. Two reviewers (HY and JT) check each other’s extracted data, and a third reviewer (HL) will assess and resolve any conflicts.

### 2.4 Risk of bias assessment

Two investigators (HL and SP) independently used the Cochrane Risk of Bias Assessment Tool (Higgins et al., 2022) to assess the quality of the included RCTs and determined “low risk of bias,” “high risk of bias,” and “unclear” for six types of bias, including selection bias, implementation bias, measurement bias, follow−up bias, reporting bias, and other biases.

### 2.5 Data analysis

Review Manager 5.4 software was used for data analysis. This study was assessed using the weighted mean differences (WMD) and 95% confidence interval (CI) for continuous type variables. The X^2^ test and I^2^ test responded to the heterogeneity of the data. If heterogeneity (I^2^ > 50%, *p* < 0.05) exists, a random−effects model is used; conversely, a fixed−effects model is used. When heterogeneity exists, we will explore the sources of heterogeneity through subgroup analysis. In addition, the funnel plot detected publication bias, and Egger’s test of Stata V14.0 was performed.

### 2.6 Sensitivity analysis

Sensitivity analysis was performed using Stata V14.0 to exclude the effect of a single RCT on the remaining study results. The robustness and reliability of the results were good if there was no significant difference before and after the exclusion of individual trials.

## 3 Results

### 3.1 Literature selection

**FIGURE 1:**
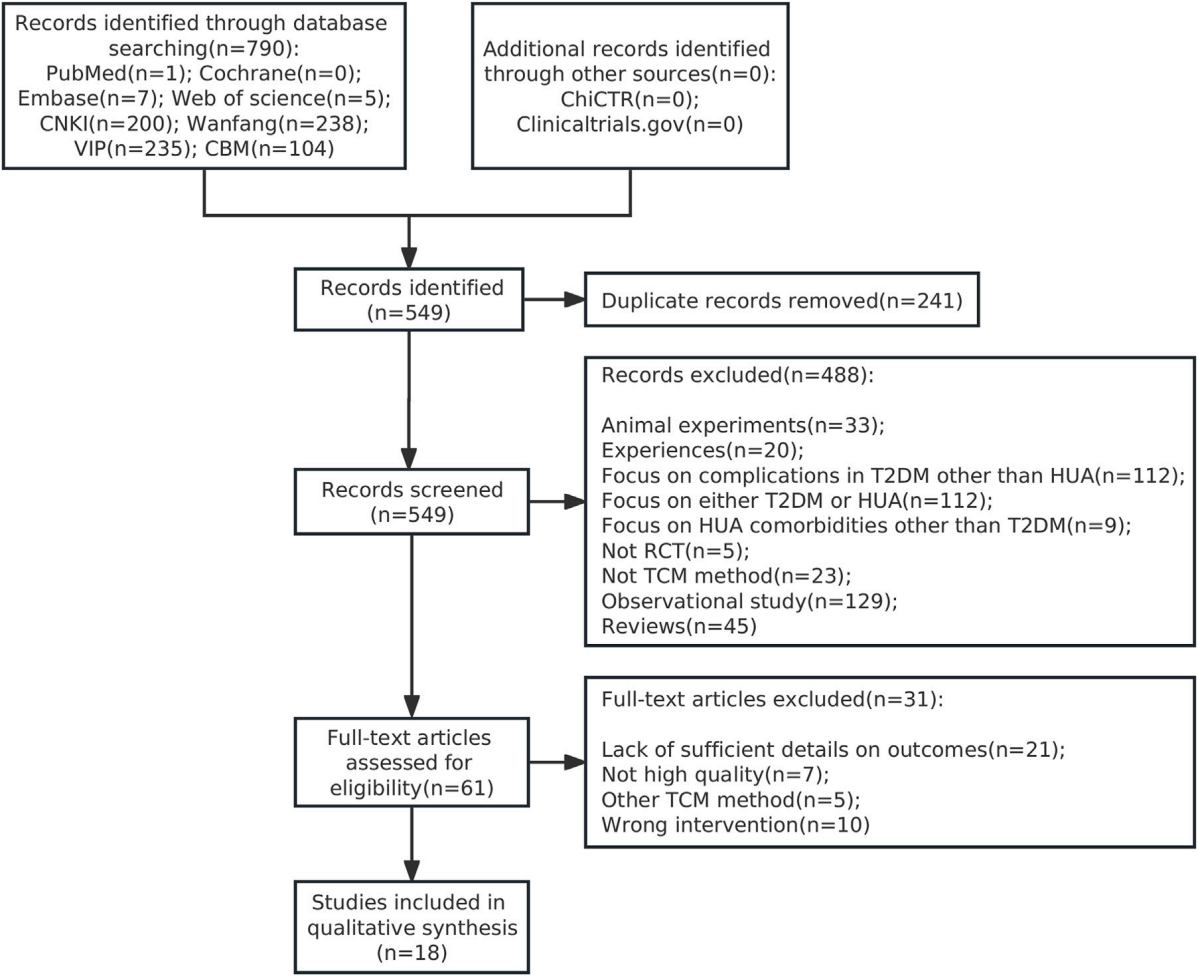
Flow diagram of studies selection process.

Based on the pre−defined search strategy, we initially screened 790 documents, 241 of which were duplicates. After excluding duplicates, 488 non−conforming articles were excluded according to their titles and abstracts. The full text of the 61 remaining articles was read, and 31 were excluded for the following reasons: lack of outcome details (n = 21), not high−quality articles (n = 7), use of other TCM therapies (n = 5), and inconsistent interventions (n = 10). 18 RCTs (Yu, 2010; Peng et al., 2014; Tang, 2014; Wu, 2015; Yin, 2015; Lou, 2017; Tang et al., 2017; Zhou, 2018; Hou, 2019; Hu and Qu, 2019; Liu, 2020; Ma et al., 2020; Shao, 2020; Gu et al., 2021; Liu, 2021; Xie et al., 2021; Zhang et al., 2021; Zou and Jiang, 2021) were eventually included for systematic evaluation. The flow diagram of the selection process is shown in Figure 1.

### 3.2 Study characteristics

A total of 18 RCTs of CHM for T2DM with HUA conducted in China from 2014 to 2021 were included in this paper, involving a total of 1,311 patients, including 657 in the treatment group and 654 in the control group. Among the 18 included studies, the shortest intervention duration was 2 weeks (Gu et al., 2021), and the longest was 3 months (Zhou, 2018; Zhang et al., 2021). Looking at the interventions in the control group included in the study, only three studies (Yu, 2010; Yin, 2015; Liu, 2020) treated with glucose−lowering drugs alone, while 15 studies (Peng et al., 2014; Tang, 2014; Wu, 2015; Lou, 2017; Tang et al., 2017; Zhou, 2018; Ma et al., 2020; Shao, 2020; Liu, 2021; Xie et al., 2021; Zhang et al., 2021; Zou and Jiang, 2021) used a combination of glucose−lowering drugs and uric acid−lowering drugs. Table 1 summarizes the characteristics of the 18 RCTs, and Supplementary Table S2 lists specific information on CHM.

**TABLE 1:** Characteristics of the included studies.

Study	Sample size	Age		Gender (M/F)		Course of disease		Co.− intervention	Intervention		Duration	Outcome
	(T/C)	T	C	T	C	T	C		T	C		
Gu et al. (2021)	36/36	53.06 ± 4.71	52.21 ± 4.85	31/5	29/7	NR	NR	Lifestyle intervention	CHM formula + conventional treatment and indometacin enteric−coated tablets (25 mg,bid)	Conventional treatment (oral hypoglycemic drug and insulin) and indometacin enteric−coated tablets (25 mg,bid)	2w	①②③④⑤⑥
Hou (2019)	30/30	74.97 ± 5.88	75.53 ± 5.2	16/14	15/15	NR	NR	Lifestyle intervention	CHM decoction + conventional treatment (hypoglycemic drug) and allopurinol tablets (100 mg,qd)	Conventional treatment (hypoglycemic drug) and allopurinol tablets (100 mg,qd)	1 m	①②④
Hu and Qu. (2019)	42/42	53.14 ± 7.02	52.29 ± 6.48	24/18	25/17	9.35 ± 2.81y	8.68 ± 2.7y	Lifestyle intervention	CHM decoction + conventional treatment, benzbromarone tablets (50 mg,qd) and sodiumbicarbonate tablets (1 g,tid)	Conventional treatment (oral hypoglycemic drug), benzbromarone tablets (50 mg,qd) and sodiumbicarbonate tablets (1 g,tid)	1 m	①③④⑤⑥
Liu (2020)	40/40	44.7 ± 6.1	44.8 ± 6.8	31/9	30/10	8.2 ± 4.2 m	8.16 ± 4.1 m	NR	CHM powder + metformin (1 g,bid)	Metformin (1 g,bid)	1 m	①②④
Liu (2021)	49/48	50.39 ± 1.5	50.37 ± 1.48	22/27	23/25	6.09 ± 0.55y	6.1 ± 0.56y	NR	CHM decoction + conventional treatment, benzbromarone tablets (50 mg,bid) and sodiumbicarbonate tablets (1 g,tid)	Conventional treatment (hypoglycemic drug and hypolipidemic drug), benzbromarone tablets (50 mg,bid) and sodiumbicarbonate tablets (1 g,tid)	1 m	①②④
Lou (2017)	30/30	57.93 ± 12.82	56.27 ± 11.28	12/18	14/16	NR	NR	Lifestyle intervention	CHM decoction + conventional treatment and benzbromarone tablets (50 mg,qd)	Conventional treatment (oral hypoglycemic drug and insulin) and benzbromarone tablets (50 mg,qd)	2 m	①②③④⑦
Ma et al. (2020)	34/34	45.08 ± 4.17	45.21 ± 3.54	29/5	27/7	3.68 ± 0.47y	3.71 ± 0.52y	NR	CHM formula + conventional treatment and colchicine tablets (1 mg,tid)	Conventional treatment (hypoglycemic drug and insulin) and colchicine tablets (1 mg,tid)	1 m	①②④⑤⑥⑦
Peng et al. (2014)	49/49	50.25 ± 2.18		41/58		9.86 ± 2.25y		Lifestyle intervention	CHM decoction + conventional treatment, pioglitazone hydrochloride tablets (15 mg,qd) and allopurinol sustained−reease capsules (250 mg,qd)	Conventional treatment (hypoglycemic drug and antihypertensive drug),pioglitazone hydrochloride tablets (15 mg,qd) and allopurinol sustained−reease capsules (250 mg,qd)	2 m	①②④⑤⑥⑦
Shao (2020)	30/30	NR	NR	16/14	15/15	6.47 ± 2.81y	6.23 ± 2.79y	Lifestyle intervention	CHM formula + conventional treatment and benzbromarone tablets (50 mg,qd)	Conventional treatment (oral hypoglycemic drug and insulin) and benzbromarone tablets (50 mg,qd)	1 m	①②③④⑦
Tang (2014)	30/30	54.2 ± 4.67	55.1 ± 8.07	16/14	17/13	6.4 ± 5.96y	5.67 ± 4.63y	Lifestyle intervention	CHM decoction + conventional treatment and allopurinol tablets (0.1 g,qd)	Conventional treatment (oral hypoglycemic drug and insulin) and allopurinol tablets (0.1 g,qd)	2 m	①②③④⑤⑥
Tang et al. (2017)	30/30	66.2 ± 10.4	67.5 ± 9.1	23/7	26/4	NR	NR	NR	CHM formula + benzbromarone tablets (50 mg,qd) and sodiumbicarbonate tablets (1 g,tid)	Benzbromarone tablets (50 mg,qd) and sodiumbicarbonate tablets (1 g,tid)	2 m	①④⑤⑥⑦
Wu (2015)	30/30	42.5 ± 5.6		31/29		3.5 ± 1.1y		Lifestyle intervention	CHM decoction + conventional treatment (hypoglycemic drug) and allopurinol tablets (0.1 g,tid)	Conventional treatment (hypoglycemic drug) and allopurinol tablets (0.1 g,tid)	1 m	①②④⑥
Xie et al. (2021)	30/30	48.71 ± 8.24	47.06 ± 6.43	26/4	28/2	0.42–12y	0.5–10y	Lifestyle intervention	CHM decoction + benzbromarone tablets (50 mg,qd) and metformin sustained−release tablets (0.5 g,bid)	Benzbromarone tablets (50 mg,qd) and metformin sustained−release tablets (0.5 g,bid)	2 m	①④⑦
Yin, (2015)	30/30	58.8 ± 10.93	58.7 ± 9.01	17/13	16/14	9.57 ± 6.09y	8.8 ± 5.58y	Lifestyle intervention	CHM decoction + conventional treatment	Conventional treatment (oral hypoglycemic drug and insulin)	2 m	①②③④⑤⑥⑦
Yu (2010)	52/50	NR	NR	30/22	28/22	5.6 ± 3.8y	5.2 ± 4.1y	NR	CHM decoction + glimepiride (2.5 mg,bid)	Glimepiride (2.5 mg,bid)	2 m	①②④⑦
Zhang et al. (2021)	50/50	47.2 ± 9.95	46.86 ± 10.1	41/9	39/11	7.36 ± 4.98y	7.46 ± 4.86y	Lifestyle intervention	CHM granule + conventional treatment, benzbromarone tablets (50 mg,qd) and sodiumbicarbonate tablets (1 g,tid)	Conventional treatment (oral hypoglycemic drug and antihypertensive drug), benzbromarone tablets (50 mg,qd) and sodiumbicarbonate tablets (1 g,tid)	3 m	①②③④⑤⑥
Zhou (2018)	32/31	56.77 ± 15.46	57.43 ± 14.15	17/13	16/14	9.63 ± 5.76y	9.57 ± 6.03y	Lifestyle intervention	CHM decoction + conventional treatment and benzbromarone tablets (50 mg,qd)	Conventional treatment (oral hypoglycemic drug and insulin) and benzbromarone tablets (50 mg,qd)	3 m	①②③④⑦
Zou (2021)	35/35	58.12 ± 5.02	58.69 ± 5.01	17/18	20/15	5.33 ± 0.14y	5.69 ± 0.14y	NR	CHM decoction + metformin hydrochloride enteric−coated tablets (0.25 g,tid),Feinuobeite (0.2 g,qd) and allopurinol tablets (50 mg,qd)	Metformin hydrochloride enteric−coated tablets (0.25 g,tid),Feinuobeite (0.2 g,qd) and allopurinol tablets (50 mg,qd)	2 m	①②③④⑤⑥

Abbreviations: T, treatment group; C,, control group; CHM, chinese herbal medicine; F, female; M, male; NR, not reported; Y, year; W, week; ①, fasting blood glucose; ②, 2−h postprandial glucose; ③, glycosylated hemoglobin; ④, uric acid; ⑤, triglyceride; ⑥, total cholesterol; ⑦, overall effective rate.

### 3.3 Risk of bias

**FIGURE 2:**
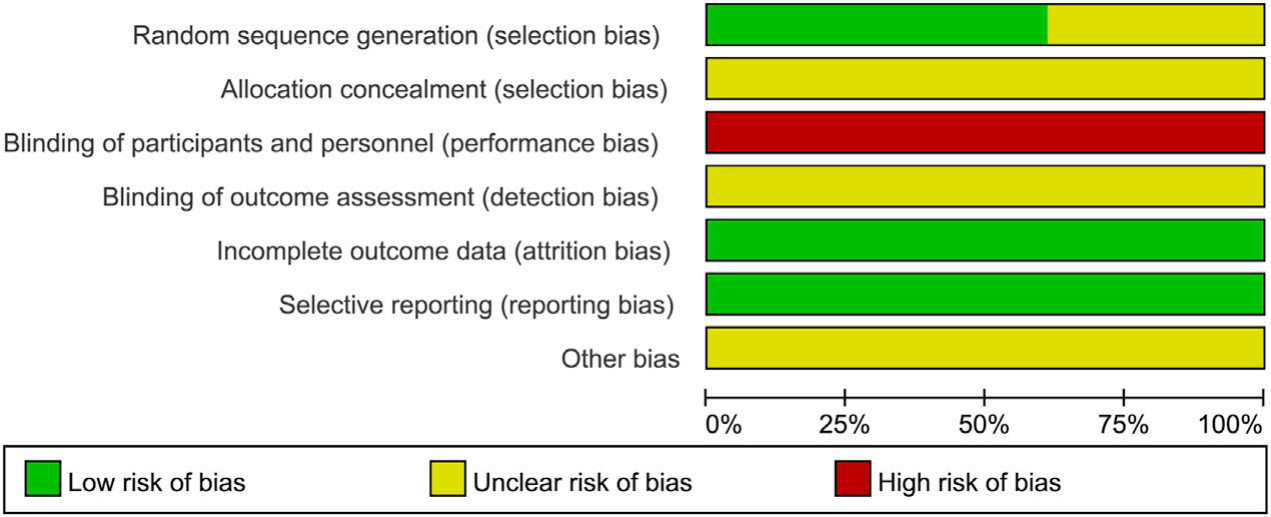
Risk of bias graph.

**FIGURE 3:**
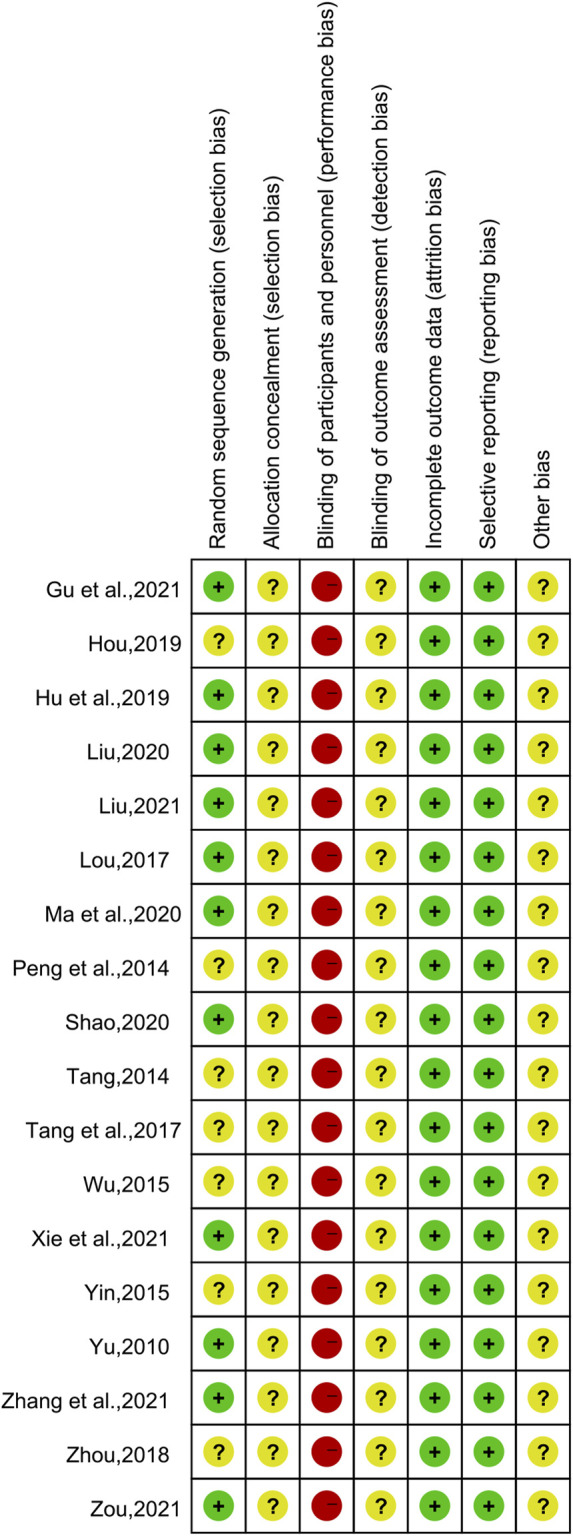
Risk of bias summary.

Of the 18 included studies, 11 studies (Yu, 2010; Lou, 2017; Hu and Qu, 2019; Liu, 2020; Ma et al., 2020; Shao, 2020; Gu et al., 2021; Liu, 2021; Xie et al., 2021; Zhang et al., 2021; Zou and Jiang, 2021) used the random number table method to generate random sequences and were at low risk of bias, while the remaining seven studies (Peng et al., 2014; Tang, 2014; Wu, 2015; Yin, 2015; Tang et al., 2017; Zhou, 2018; Hou, 2019) had unclear randomization and were at “unclear risk.” None of the studies demonstrated participant or study staff blindness and were therefore assessed as high risk of bias. All studies were assessed as low risk of bias in terms of incomplete data and reporting bias. All studies had unclear allocation concealment and did not have insufficient information to report other biases and were therefore assessed as “unclear risk.” The results of the risk bias assessment for the included studies are shown in Figure 2 and Figure 3.

### 3.4 Effects of CHM on glucose metabolism

#### 3.4.1 FBG

**FIGURE 4:**
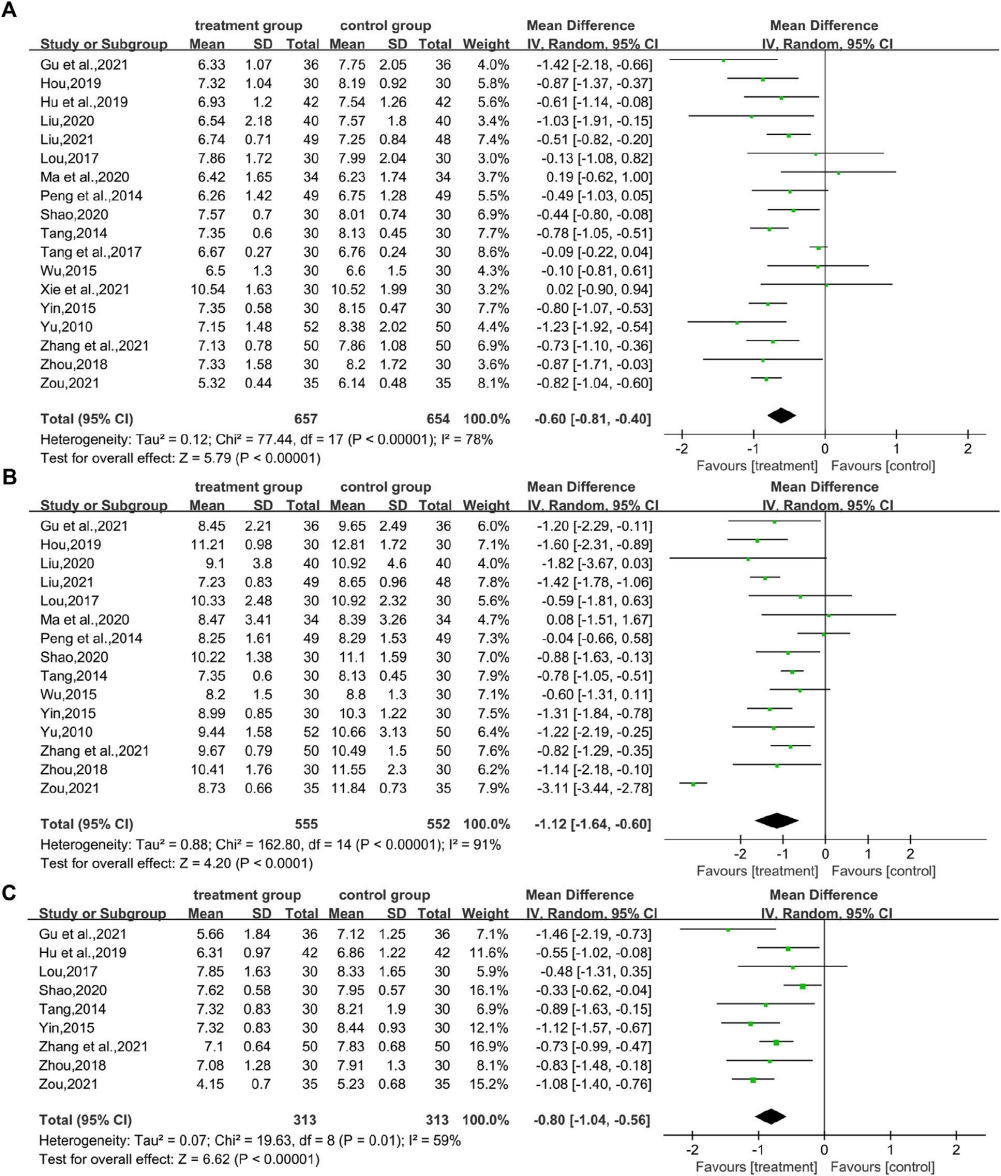
Forest plot for glucose metabolism. **(A)** FBG **(B)** 2hPG; and **(C)** HbA1c.

In total, 18 studies (Yu, 2010; Peng et al., 2014; Tang, 2014; Wu, 2015; Lou, 2017; Tang et al., 2017; Zhou, 2018; Hou, 2019; Hu and Qu, 2019; Liu, 2020; Ma et al., 2020; Shao, 2020; Gu et al., 2021; Liu, 2021; Xie et al., 2021; Zhang et al., 2021; Zou and Jiang, 2021) reported FBG levels in a total of 1,311 patients. The analysis showed that the combination of CHM and WM was effective in improving FBG levels in T2DM with HUA with statistical significance (WMD = −0.60.95% CI [−0.81, −0.40], *p* < 0.00001, I^2^ = 78, high heterogeneity, random effect model; Figure 4). In subgroup analysis results showed no differences between different intervention durations (*p* = 0.12), and different control treatments (*p* = 0.05), while differences were found between different types of drugs (*p* = 0.03). (Table 2; Supplementary Figure S1).

**TABLE 2:** Subgroup analysis for outcomes.

	Number of comparisons	Result	*p*−value for overall effect	I2 (%)	*p*−value for subgroup difference
FBG		WMD/RR (95%CI)			
All comparisons	18	−0.60 [−0.81, −0.40]	<0.00001	78	
Intervention duration					0.12
2w	1	−1.42 [−2.18, −0.66]	0.0002	−	
1 m	7	−0.51 [−0.73, −0.29]	<0.00001	23	
2 m	8	−0.58 [−0.91, −0.24]	0.0008	88	
3 m	2	−0.75 [−1.09, −0.42]	<0.0001	0	
Types of drugs					0.03
Benzbromarone tablets	8	−0.42 [−0.67, −0.18]	0.00009	66	
Other drugs	10	−0.76 [−0.95, −0.58]	<0.00001	41	
Different control treatment					0.05
Hypoglycemic therapy	3	−0.87 [−1.11, −0.63]	<0.00001	0	
Hypoglycemic therapy and uric acid lowering therapy	15	−0.54 [−0.76, −0.32]	<0.00001	78	
2hPG					
All comparisons	15	−1.12 [−1.64, −0.60]	<0.0001	91	
Intervention duration					0.83
2w	1	−1.20 [−2.29, −0.11]	0.03	−	
1 m	6	−1.12 [−1.55, −0.70]	<0.00001	44	
2 m	6	−1.20 [−2.28, −0.11]	0.03	97	
3 m	2	−0.87 [−1.30, −0.45]	<0.0001	0	
Types of drugs					0.82
Benzbromarone tablets	5	−1.08 [−1.41, −0.76]	<0.00001	26	
Other drugs	10	−1.18 [−1.96, −0.40]	0.003	94	
Different control treatment					0.5
Hypoglycemic therapy	3	−1.32 [−1.77, −0.87]	<0.00001	0	
Hypoglycemic therapy and uric acid lowering therapy	12	−1.06 [−1.67, −0.44]	0.0008	93	
HbA1c					
All comparisons	9	−0.80 [−1.04, −0.56]	<0.00001	59	
Intervention duration					0.0008
2w	1	−1.46 [−2.19, −0.73]	<0.0001	−	
1 m	2	−0.39 [−0.64, −0.14]	0.002	0	
2 m	4	−1.02 [−1.26, −0.79]	<0.00001	0	
3 m	2	−0.74 [−0.98, −0.50]	<0.00001	0	
Types of drugs					0.0005
Benzbromarone tablets	5	−0.57 [−0.76, −0.37]	<0.00001	16	
Other drugs	4	−1.11 [−1.35, −0.88]	<0.00001	0	
Different control treatment					0.16
Hypoglycemic therapy	1	−1.12 [−1.57, −0.67]	<0.00001	−	
Hypoglycemic therapy and uric acid lowering therapy	8	−0.76 [−1.01, −0.51]	<0.00001	58	
UA					
All comparisons	18	−53.47 [−67.45, −39.48]	<0.00001	93	
Intervention duration					0.06
2w	1	−76.32 [−99.72, −52.92]	<0.00001	−	
1 m	7	−66.50 [−92.92, −40.07]	<0.00001	95	
2 m	8	−39.40 [−55.81, −23.00]	<0.00001	89	
12w	2	−54.39 [−69.68, −39.10]	<0.00001	0	
Types of drugs					0.19
Benzbromarone tablets	8	−43.31 [−58.45, −28.16]	<0.00001	87	
Other drugs	10	−61.44 [−84.03, −38.85]	<0.00001	94	
Different control treatment					0.005
Hypoglycemic therapy	3	−86.43 [−110.40, −62.47]	<0.00001	83	
Hypoglycemic therapy and uric acid lowering therapy	15	−46.60 [−60.70, −32.49]	<0.00001	92	
TG					
All comparisons	9	−0.56 [−0.74, −0.38]	<0.00001	91	
Intervention duration					<0.00001
2w	1	−1.32 [−1.63, −1.01]	<0.00001	−	
1 m	2	−0.39 [−0.71, −0.08]	0.02	27	
2 m	5	−0.52 [−0.73, −0.32]	<0.00001	92	
3 m	1	−0.35 [−0.55, −0.15]	0.0008	−	
Types of drugs					<0.00001
Benzbromarone tablets	4	−0.24 [−0.39, −0.10]	0.001	50	
Other drugs	5	−0.72 [−0.90, −0.54]	<0.00001	86	
Different control treatment					0.07
Hypoglycemic therapy	1	−0.76 [−0.90, −0.62]	<0.00001	−	
Hypoglycemic therapy and uric acid lowering therapy	8	−0.54 [−0.73, −0.34]	<0.00001	90	
TC					
All comparisons	10	−0.49 [−0.65, −0.33]	<0.00001	58	
Intervention duration					0.91
2w	1	−0.38 [−0.75, −0.01]	0.04	−	
1 m	3	−0.58 [−0.99, −0.16]	0.007	79	
2 m	5	−0.49 [−0.75, −0.24]	0.0001	62	
3 m	1	−0.45 [−0.77, −0.13]	0.007	−	
Types of drugs					0.54
Benzbromarone tablets	4	−0.41 [−0.74, −0.07]	0.02	55	
Other drugs	6	−0.53 [−0.72, −0.33]	<0.00001	65	
Different control treatment					0.04
Hypoglycemic therapy	1	−0.83 [−1.15, −0.51]	<0.00001	−	
Hypoglycemic therapy and uric acid lowering therapy	9	−0.45 [−0.60, −0.30]	<0.00001	48	
overall effective rate					
All comparisons	10	1.29 [1.13, 1.48]	0.0002	72	
Intervention duration					0.39
1 m	3	1.15 [0.98, 1.36]	0.1	53	
2 m	6	1.15 [0.98, 1.36]	0.005	80	
3 m	1	1.35 [0.96, 1.89]	0.08	−	
Types of drugs					0.53
Benzbromarone tablets	5	1.36 [1.20, 1.55]	<0.00001	0	
Other drugs	5	1.26 [1.01, 1.56]	0.04	85	
Different control treatment					0.21
Hypoglycemic therapy	3	1.47 [1.13, 1.90]	0.003	64	
Hypoglycemic therapy and uric acid lowering therapy	7	1.22 [1.06, 1.39]	0.005	62	

#### 3.4.2 2hPG

In total, 15 studies (Yu, 2010; Peng et al., 2014; Tang, 2014; Wu, 2015; Lou, 2017; Zhou, 2018; Hou, 2019; Liu, 2020; Ma et al., 2020; Shao, 2020; Gu et al., 2021; Liu, 2021; Zhang et al., 2021; Zou and Jiang, 2021) evaluated 2hPG levels, involving a total of 1,107 patients. The overall analysis showed that the combination of CHM and WM improved the 2hPG level in T2DM with HUA better than WM alone with statistical significance (WMD = −1.12.95% CI [−1.64, −0.60], *p* < 0.0001, I^2^ = 91, high heterogeneity, random effect model; Figure 4). In the results of the subgroup analysis, there were no differences between the different intervention durations, different types of drugs, and different control treatments (*p* = 0.83, 0.82, and 0.5, respectively). (Table 2; Supplementary Figure S1).

#### 3.4.3 HbA1c

A total of nine studies (involving a total of 626 patients) (Tang, 2014; Yin, 2015; Lou, 2017; Zhou, 2018; Hu and Qu, 2019; Shao, 2020; Gu et al., 2021; Zhang et al., 2021; Zou and Jiang, 2021) reported on HbA1c levels in patients with T2DM with HUA in CHM. The results showed that the combination of CHM and WM had a statistically significant reduction in HbA1c levels in T2DM with HUA (WMD = −0.80.95% CI [−1.04, −0.56], *p* < 0.00001, I^2^ = 59, high heterogeneity, random effect model; Figure 4). Subgroup analysis showed differences by intervention durations (*p* = 0.0008) and by types of drugs (*p* = 0.0005), while there were no differences by control treatments (*p* = 0.16). (Table 2; Supplementary Figure S1).

### 3.5 Effect of CHM on uric acid

**FIGURE 5:**
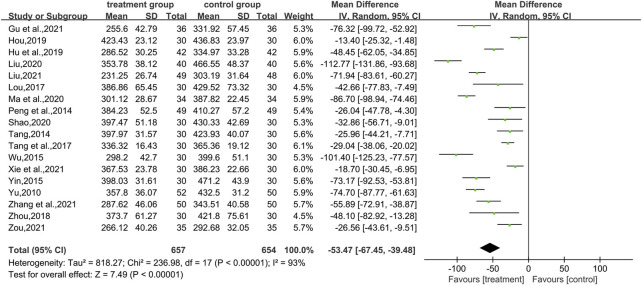
Forest plot for uric acid.

A total of 18 studies (Yu, 2010; Peng et al., 2014; Tang, 2014; Wu, 2015; Lou, 2017; Tang et al., 2017; Zhou, 2018; Hou, 2019; Hu and Qu, 2019; Liu, 2020; Ma et al., 2020; Shao, 2020; Gu et al., 2021; Liu, 2021; Xie et al., 2021; Zhang et al., 2021; Zou and Jiang, 2021) used uric acid levels as an outcome indicator, involving a total of 1,311 patients. In terms of improving uric acid levels, the combination of CHM and WM was statistically more effective (WMD = −53.47.95% CI [−67.45, −39.48], *p* < 0.00001, I^2^ = 93, high heterogeneity, random effects model; Figure 5). The results of the subgroup analysis showed no differences between the different intervention durations (*p* = 0.06), between the different types of drugs (*p* = 0.19), and differences between the different control treatments (*p* = 0.005). (Table 2; Supplementary Figure S1).

### 3.6 Effects of CHM on blood lipids

#### 3.6.1 TG

**FIGURE 6:**
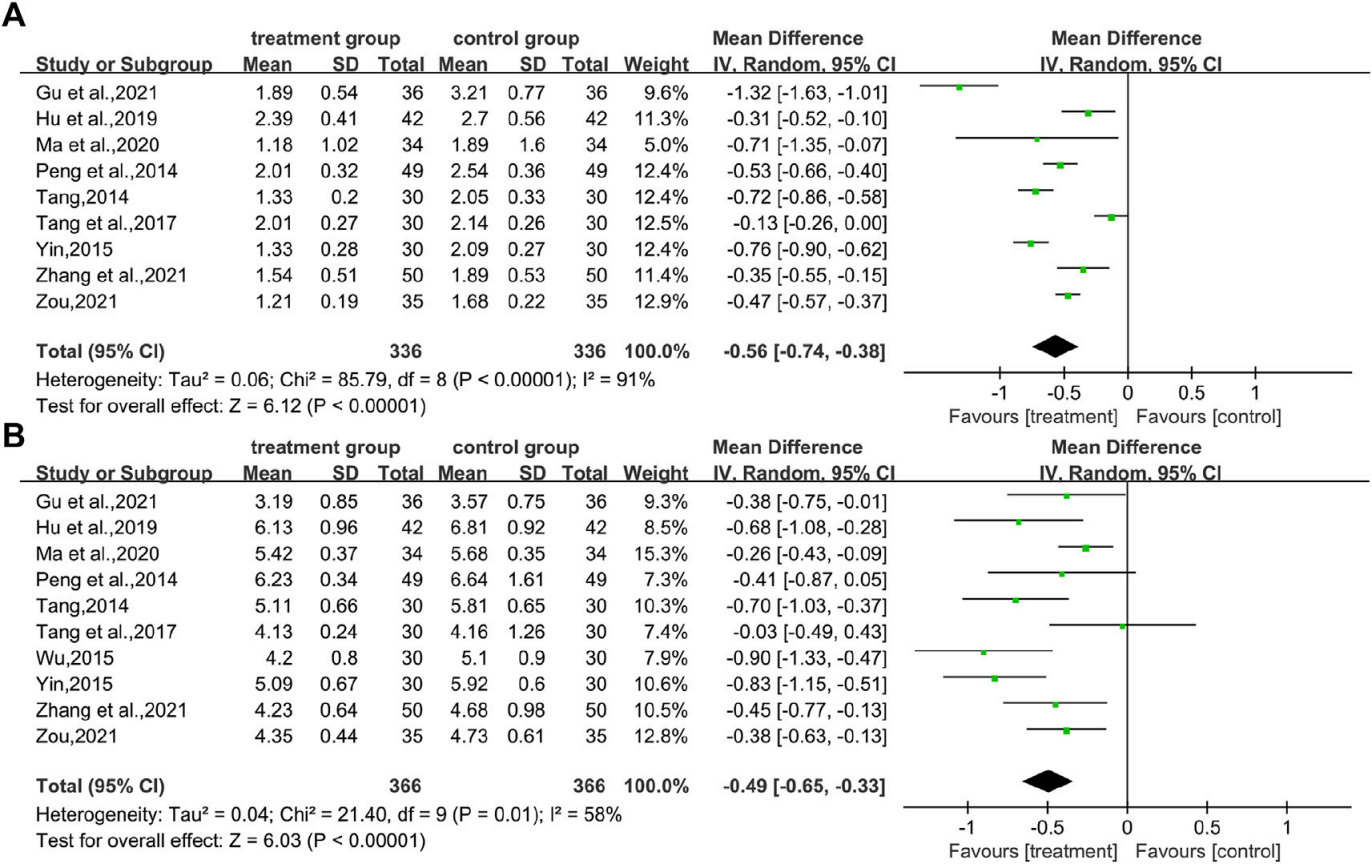
Forest plot for lipid indices. **(A)** TG; and **(B)** TC.

In total, nine studies (Peng et al., 2014; Tang, 2014; Yin, 2015; Tang et al., 2017; Hu and Qu, 2019; Ma et al., 2020; Gu et al., 2021; Zhang et al., 2021; Zou and Jiang, 2021) reported TG levels, covering 672 patients. There was a difference between the two groups (WMD = −0.56.95% CI [−0.74, −0.38], *p* < 0.00001, I^2^ = 91, high heterogeneity, random effects model; Figure 6). It indicates that the combination of CHM and WM effectively improved TG levels in patients with T2DM with HUA, which was statistically significant. Regarding the subgroup analysis, differences were found by intervention durations (*p* < 0.00001) and by types of drugs (*p* < 0.00001), while there were no differences by control treatments (*p* = 0.07). (Table 2; Supplementary Figure S1).

#### 3.6.2 TC

The TC index was mentioned in 10 studies (Peng et al., 2014; Tang, 2014; Wu, 2015; Yin, 2015; Tang et al., 2017; Hu and Qu, 2019; Ma et al., 2020; Gu et al., 2021; Zhang et al., 2021; Zou and Jiang, 2021) involving a total of 732 patients. The analysis differed between the two groups (WMD = −0.49.95% CI [−0.65, −0.33], *p* < 0.00001, I^2^ = 58, high heterogeneity, random effects model; Figure 6). In terms of subgroup analysis, there were no differences by intervention durations (*p* = 0.91) and by types of drugs (*p* = 0.54), while differences existed by control treatments (*p* = 0.04). (Table 2; Supplementary Figure S1).

### 3.7 Overall effective rate and adverse effects

#### 3.7.1 Overall effective rate

**FIGURE 7:**
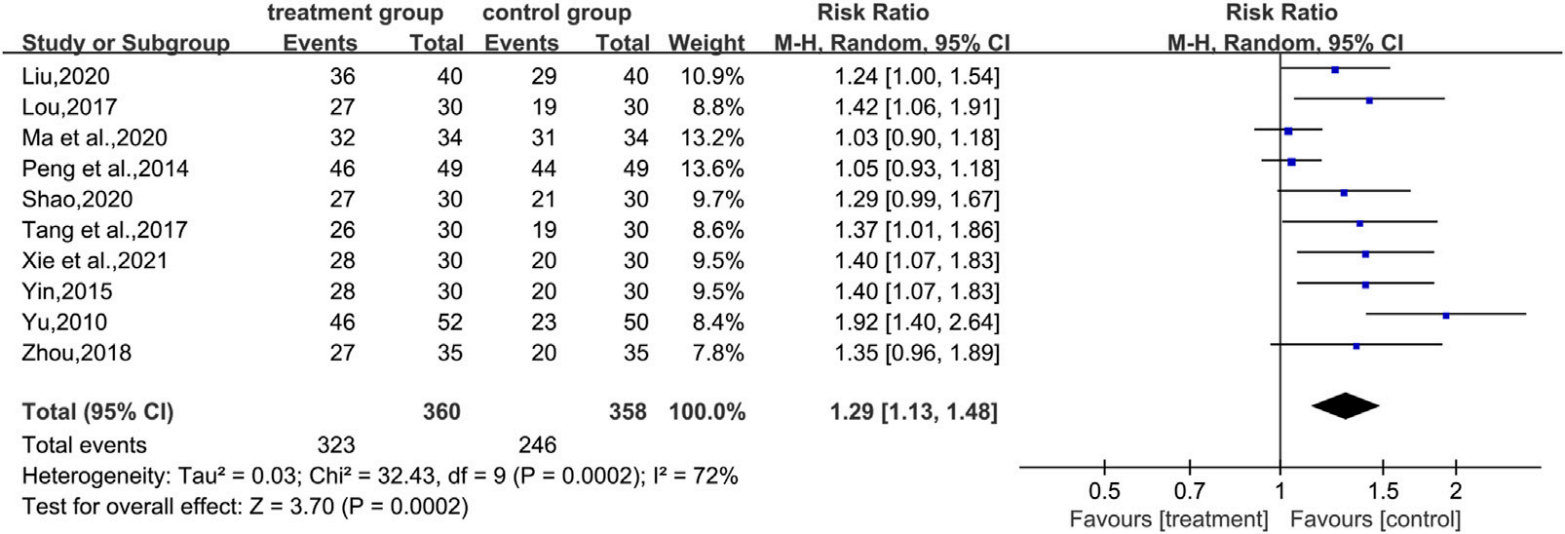
Forest plot for overall effective rate.

In total, 10 studies (Yu, 2010; Peng et al., 2014; Yin, 2015; Lou, 2017; Tang et al., 2017; Zhou, 2018; Liu, 2020; Ma et al., 2020; Shao, 2020; Xie et al., 2021) reported total effective rates, involving a total of 718 patients. The analysis showed that the combination of CHM and WM effectively improved the overall effective rate of T2DM with HUA in a statistically significant manner compared with WM alone (RR = 1.29.95% CI [1.13, 1.48], *p* = 0.0002, I^2^ = 72%, high heterogeneity, random effects model; Figure 7). In the results of the subgroup analysis, there were no differences between the different intervention durations, different types of drugs, and different control treatments (*p* = 0.39, 0.53, and 0.21, respectively). (Table 2; Supplementary Figure S1).

#### 3.7.2 Adverse effects

Adverse effects were mentioned in nine studies. Six of these studies (Yu, 2010; Yin, 2015; Zhou, 2018; Hou, 2019; Ma et al., 2020; Shao, 2020) showed no adverse events in patients while taking the drug, and three other studies (Hu and Qu, 2019; Liu, 2021; Zhang et al., 2021) reported adverse events. The most common adverse reactions that occur when combining CHM and WM are gastrointestinal discomfort, such as bloating and diarrhea; skin abnormalities such as itching and rash may also occur.

### 3.8 Publication bias

**FIGURE 8:**
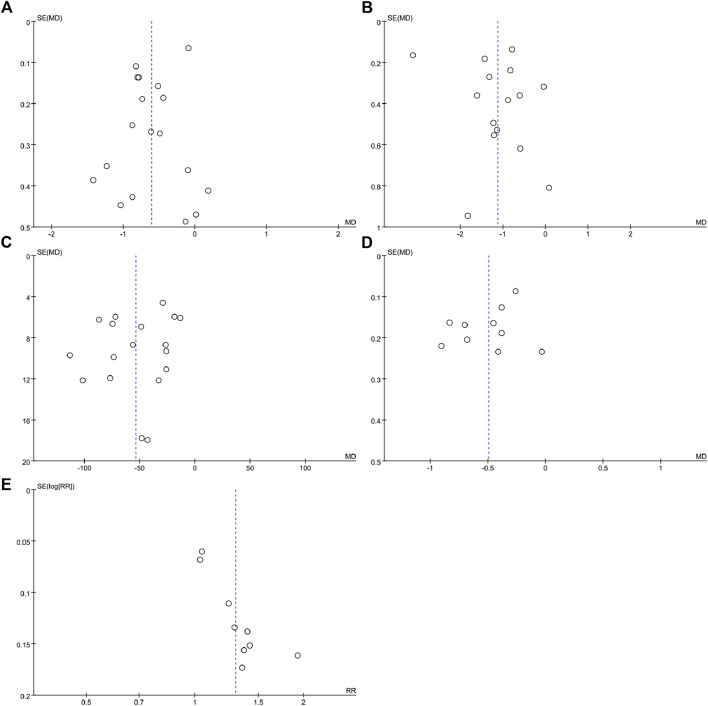
Funnel plots for assessing publication bias. **(A)** FBG **(B)** 2hPG **(C)** UA **(D)** TC; and **(E)** overall effective rate.

Results from ten or more included studies were assessed for publication bias. Publication bias needs to be assessed by funnel plot and Egger’s test. There was a slight asymmetry in the funnel plots (Figures 8B,C,E) and Egger’s test indicated possible publication bias for 2hPG (t = −3.95, *p* = 0.002), UA (t = −3.21, *p* = 0.005) and overall effective rate (t = 6.51 *p* < 0.0001) (Supplementary Figure S2). While the rest of the funnel plots (Figures 8A,D) showed symmetrical funnel plots, Egger’s test indicated no publication bias for FBG (t = −1.36, *p* = 0.191) and TC (t = −2.09, *p* = 0.07) (Supplementary Figure S2).

### 3.9 Sensitivity analysis

To ensure the robustness and reliability of the analysis results, sensitivity analysis was performed using Stata V14.0. No significant changes in the results of the remaining trials were observed after the exclusion of each study, indicating that the results were robust and reliable (Supplementary Figure S3).

## 4 Discussion

### 4.1 Research results

A total of 790 publications were retrieved for this study, and 18 RCTs involving 1,311 patients were finally included after a detailed review of the literature. This systematic evaluation and meta−analysis assessed the efficacy and safety of CHM in the treatment of T2DM with HUA. The results of the study showed that the combination of CHM and WM was more effective in treating patients with T2DM with HUA than WM alone, with significant improvements in glucose metabolism (FBG, 2hPG, HbA1c), UA, and lipids (TG, TC). The high heterogeneity present in the data analysis stemmed from differences in interventions. The sources of heterogeneity were examined by subgroup analysis, using different intervention durations, different types of drugs, and different control treatments to explain the effect of heterogeneity. In the literature assessing safety, most of the adverse reactions were not reported, and a few had non−serious adverse reactions. Funnel plot analysis and Egger’s test revealed possible publication bias for some indicators. The heterogeneity of this study is high and the methodological quality is low, but sensitivity analysis indicates that the results are robust.

### 4.2 Quality of evidence

We used GRADEpro to describe the quality of evidence for the results. The results, assessed according to study limitations, inconsistencies, indirectness, imprecision, and publication bias, indicated that the quality of evidence was low for all outcomes (Table 3). The low quality of evidence is mainly due to the low methodological quality and high heterogeneity. Therefore, further assessment of clinical effectiveness and safety through large−scale, high−quality, comprehensive, and standardized RCT results is needed.

**TABLE 3:** Certainty of evidence: CHM compared to control treatment for T2DM with HUA.

Certainty assessment							No. Of patients		Effect		Certainty	Importance
No.Of studies	Study design	Risk of bias	Inconsistency	Indirectness	Imprecision	Other considerations	CHM	Control treatment	Relative (95% CI)	Absolute (95% CI)		
Fasting blood glucose18												
18	Randomized trials	Serious a	Serious b	Not serious	Not serious	None	657	654	−	MD 0.6 lower (0.81–0.4 lower)	⊕⊕⊚⊚Low	Important
2−h postprandial glucose												
15	Randomized trials	Serious a	Serious b	Not serious	Not serious	None	555	552	−	MD 1.12 lower (1.64–0.6 lower)	⊕⊕⊚⊚Low	Important
glycosylated hemoglobin												
9	Randomized trials	Serious a	Serious b	Not serious	Not serious	None	313	313	−	MD 0.8 lower (1.04–0.56 lower)	⊕⊕⊚⊚Low	Important
uric acid												
18	Randomized trials	Serious a	Serious b	Not serious	Not serious	None	657	654	−	MD 53.47 lower (67.45–39.48 lower)	⊕⊕⊚⊚Low	Important
triglyceride												
9	Randomized trials	Serious a	Serious b	Not serious	Not serious	None	336	336	−	MD 0.56 lower (0.74–0.38 lower)	⊕⊕⊚⊚Low	Important
total cholesterol												
10	Randomized trials	Serious a	Serious b	Not serious	Not serious	None	366	366	−	MD 0.49 lower (0.65–0.33 lower)	⊕⊕⊚⊚Low	Important
overall effective rate												
10	Randomized trials	Serious a	Serious b	Not serious	Not serious	None	323/360 (89.7%)	246/358 (68.7%)	RR 1.29 (1.13–1.48)	199 more per 1,000 (from 89 more to 330 more)	⊕⊕⊚⊚Low	Important

Abbreviations: CI, confidence interval; MD, mean difference; RR, risk ratio; a, the risk of bias assessment is mostly “unclear risk” in articles; b, here is serious heterogeneity among the studies included in the analysis of this outcome.

### 4.3 Frequency of Chinese herb medicines

**70** single CHMs were recorded in the 18 RCTs included in this study, and those with more than five occurrences are listed in the table in descending order of frequency (Table 4). The most frequently occurring CHMs was *Dioscorea septemloba* Thunb (*bì xiè*), which appeared 11 times. The results of the study of the pharmacological effects of *Dioscorea septemloba* Thunb. Showed that the total saponin of *Dioscorea* is its main component and the primary substance that exerts its pharmacological activity (Chen et al., 2017), with hyporheic acid, renal protection, and anti−inflammatory and immunomodulatory effects (Xiao and Li, 2018). Total saponin of *Dioscorea* reduces uric acid reabsorption by down−regulating the expression of renal tubular uric acid transporter one and up−regulates the expression of organic anion transporter, thereby increasing uric acid excretion (Chen et al., 2013; Zhu, 2013; Chen et al., 2015); total saponin of *Dioscorea* attenuates sodium urate−induced inflammatory response and improves insulin resistance (Li G. Y. et al., 2020; Guoying et al., 2021). Other CHMs such as *Astragalus mongholicus* Bunge also have related pharmacological effects. *Astragalus mongholicus* Bunge has a complex chemical composition with a high content of flavonoids, polysaccharides, and saponins, which are the main active components of *Astragalus* (Zhang S. J. et al., 2022). *Astragalus* polysaccharide can exert hypoglycemic effects by upregulating the expression of glucose transporter protein four mRNA; in addition, astragaloside inhibits hepatic gluconeogenesis by regulating the PI3K/Akt/FoxO1 signaling pathway to regulate blood glucose, while protecting against the damage caused to pancreatic β−cells after activation of uric acid by this pathway, and also effectively improves insulin resistance by inhibiting protein tyrosine phosphatase 1B in insulin−resistant human hepatocytes HepG2 cells (Dai et al., 2022; Jiang et al., 2022). In terms of improving uric acid levels, astragalus polysaccharides achieve inhibition of uric acid production by inhibiting xanthine oxidase activity (Li et al., 2021).

**TABLE 4:** Frequency of CHM (more than 5 times).

NO.	Chinese herb	Latin name	Frequency
1	Bi xie (bì xiè)	*Dioscorea septemloba* Thunb. [Dioscoreaceae; Dioscoreae hypoglaucae rhizoma]	11
2	Huang Qi (huáng qí)	*Astragalus mongholicus* Bunge [Fabaceae; Astragali radix]	9
3	Da Huang (dà huáng)	*Rheum palmatum* L. [Polygonaceae; Rhei radix et rhizoma]	9
4	Tu Fu Ling (tǔ fú líng)	*Smilax glabra* Roxb. [Smilacaceae; Smilacis glabrae rhizoma]	9
5	Yi Ren (yì yǐ rén)	*Coix lacryma-jobi* L. [Poaceae; Coicis semen]	9
6	Ze Xie (zé xiè)	*Coix lacryma-jobi* L. [Poaceae; Coicis semen]	9
7	Bai Zhu (bái zhú)	*Atractylodes macrocephala* Koidz. [Asteraceae; Atractylodis macrocephalae rhizoma]	8
8	Cang Zhu (cāng zhú)	*Atractylodes lancea* (Thunb.) DC. [Asteraceae; Atractylodis rhizoma]	8
9	Dan Shen (dān shēn)	*Salvia miltiorrhiza* Bunge [Lamiaceae; Salviae miltiorrhizae radix et rhizoma]	7
10	Dang Shen (dǎng shēn)	*Codonopsis pilosula* (Franch.) Nannf. [Campanulaceae; Codonopsis radix]	7
11	Sheng Di Huang (shēng dì huáng)	*Rehmannia glutinosa* (Gaertn.) DC. [Orobanchaceae; Rehmanniae radix]	7
12	Gan Cao (gān cǎo)	*Glycyrrhiza uralensis* Fisch. ex DC. [Fabaceae; Glycyrrhizae radix et rhizoma]	6

### 4.4 Research strengths and limitations

This study is the first to focus on and evaluate CHM’s effectiveness in treating T2DM with HUA. The methods and requirements of systematic evaluation were strictly followed during the study, and GRADEpro was used to describe the quality of evidence for the results. In contrast, the results were interpreted with care. The results showed that the combination of CHM and WM treatment improved glucolipid metabolism, uric acid levels, and overall efficiency in patients with T2DM with HUA with fewer adverse effects than WM alone. Therefore, it can provide a new idea and direction for clinical treatment.

However, this study also has some limitations. First, significant heterogeneity was demonstrated in the analysis. Although subgroup analysis was performed to explain the source of the high heterogeneity, the heterogeneity may also be related to patient gender, herbal dosage form, and other reasons. Second, the quality of evidence was poor for all outcomes, and the presence of low methodological quality and high heterogeneity due to uncertainty in publication bias mostly may have affected the results. In addition, although two investigators independently performed the assessment of risk bias, there was subjectivity in the evaluation of risk bias. More, obesity is a common influencing factor for T2DM and HUA, while contributing to each other’s development through inflammatory responses. However, the present study outcome indicators did not involve body mass index and some inflammatory indicators. Finally, external intake of purine−rich foods is an essential source of HUA, making dietary management particularly important for improving T2DM with HUA. Moreover, this study could not determine the effect of health management on patients with T2DM with HUA, which provides a direction for further studies to follow.

## 5 Conclusion

In conclusion, this study shows that CHM combined with WM is effective in treating patients with T2DM with HUA and improving glucolipid metabolism and high uric acid levels, with high overall clinical efficiency. However, due to the low quality of the evidence, it should be used with caution in clinical settings. Therefore, high−quality, large−sample RCTs will be needed in the future to provide more accurate, credible, and convincing evidence for the efficacy and safety of CHM in the treatment of T2DM with HUA.

## Data Availability

The original contributions presented in the study are included in the article/Supplementary Material, further inquiries can be directed to the corresponding author.

## Abbreviations

2hPG, 2-h postprandial glucose; CHM, chinese herbal medicine; CI, confidence interval; CNKI, china national knowledge infrastructure; FBG, fasting blood glucose; GRADE, grading of recommendations assessment, development, and evaluation; HbA1c, glycated hemoglobin; HUA, hyperuricemia; PRISMA-P, preferred reporting items for systematic review and meta-analysis protocols; RCTs, randomized controlled trials; T2DM, type 2 diabetes mellitus; TC, total cholesterol; TG: triglyceride; RR, risk ratio; TCM, traditional chinese medicine; UA, uric acid; WM, western medicine; WMD, weighted mean differences.
