# Supplementary material for: Chinese herbal medicine combined with western medicine for the treatment of type 2 diabetes mellitus with hyperuricemia: A systematic review and meta-analysis
Source: Front Pharmacol. 2023 Jan 24;14:1102513. doi: 10.3389/fphar.2023.1102513 (PMC9902346; doi:10.3389/fphar.2023.1102513)
Supplement: Supplementary file 1 [file DataSheet1.docx]

Supplementary Material

# Supplementary File S1 The PRISMA checklist of this meta-analysis

| **Section/topic** | **#** | **Checklist item** | **Reported on page #** |
| --- | --- | --- | --- |
| **TITLE** | | |  |
| Title | 1 | Identify the report as a systematic review, meta-analysis, or both. | 1 |
| **ABSTRACT** | | |  |
| Structured summary | 2 | Provide a structured summary including, as applicable: background; objectives; data sources; study eligibility criteria, participants, and interventions; study appraisal and synthesis methods; results; limitations; conclusions and implications of key findings; systematic review registration number. | 2 |
| **INTRODUCTION** | | |  |
| Rationale | 3 | Describe the rationale for the review in the context of what is already known. | 3 |
| Objectives | 4 | Provide an explicit statement of questions being addressed with reference to participants, interventions, comparisons, outcomes, and study design (PICOS). | 3 |
| **METHODS** | | |  |
| Protocol and registration | 5 | Indicate if a review protocol exists, if and where it can be accessed (e.g., Web address), and, if available, provide registration information including registration number. | 2,4;  CRD4202235  1519 |
| Eligibility criteria | 6 | Specify study characteristics (e.g., PICOS, length of follow-up) and report characteristics (e.g., years considered, language, publication status) used as criteria for eligibility, giving rationale. | 4 |
| Information sources | 7 | Describe all information sources (e.g., databases with dates of coverage, contact with study authors to identify additional studies) in the search and date last searched. | 4 |
| Search | 8 | Present full electronic search strategy for at least one database, including any limits used, such that it could be repeated. | 4; Supplement Table S1 |
| Study selection | 9 | State the process for selecting studies (i.e., screening, eligibility, included in systematic review, and, if applicable, included in the meta-analysis). | 4 |
| Data collection process | 10 | Describe method of data extraction from reports (e.g., piloted forms, independently, in duplicate) and any processes for obtaining and confirming data from investigators. | 4 |
| Data items | 11 | List and define all variables for which data were sought (e.g., PICOS, funding sources) and any assumptions and simplifications made. | 4 |
| Risk of bias in individual studies | 12 | Describe methods used for assessing risk of bias of individual studies (including specification of whether this was done at the study or outcome level), and how this information is to be used in any data synthesis. | 4-5 |
| Summary measures | 13 | State the principal summary measures (e.g., risk ratio, difference in means). | 5 |
| Synthesis of results | 14 | Describe the methods of handling data and combining results of studies, if done, including measures of consistency (e.g., I^2^_)_ for each meta-analysis. | 5 |

| **Section/topic** | **#** | **Checklist item** | **Reported on page #** |
| --- | --- | --- | --- |
| Risk of bias across studies | 15 | Specify any assessment of risk of bias that may affect the cumulative evidence (e.g., publication bias, selective reporting within studies). | 5 |
| Additional analyses | 16 | Describe methods of additional analyses (e.g., sensitivity or subgroup analyses, meta-regression), if done, indicating which were pre-specified. | 5 |
| **RESULTS** |  | |  |
| Study selection | 17 | Give numbers of studies screened, assessed for eligibility, and included in the review, with reasons for exclusions at each stage, ideally with a flow diagram. | 5; Figure1 |
| Study characteristics | 18 | For each study, present characteristics for which data were extracted (e.g., study size, PICOS, follow-up period) and provide the citations. | 5;  Table1 |
| Risk of bias within studies | 19 | Present data on risk of bias of each study and, if available, any outcome level assessment (see item 12). | 5-6;  Figure2 |
| Results of individual studies | 20 | For all outcomes considered (benefits or harms), present, for each study: (a) simple summary data for each intervention group (b) effect estimates and confidence intervals, ideally with a forest plot. | 5-6;  Figure3 |
| Synthesis of results | 21 | Present results of each meta-analysis done, including confidence intervals and measures of consistency. | 6-7;  Figure4-7 |
| Risk of bias across studies | 22 | Present results of any assessment of risk of bias across studies (see Item 15). | 8;  Figure8 |
| Additional analysis | 23 | Give results of additional analyses, if done (e.g., sensitivity or subgroup analyses, meta-regression [see Item 16]). | 8 |
| **DISCUSSION** |  | |  |
| Summary of evidence | 24 | Summarize the main findings including the strength of evidence for each main outcome; consider their relevance to key groups (e.g., healthcare providers, users, and policy makers). | 8 |
| Limitations | 25 | Discuss limitations at study and outcome level (e.g., risk of bias), and at review-level (e.g., incomplete retrieval of identified research, reporting bias). | 9 |
| Conclusions | 26 | Provide a general interpretation of the results in the context of other evidence, and implications for future research. | 9-10 |
| **FUNDING** |  | |  |
| Funding | 27 | Describe sources of funding for the systematic review and other support (e.g., supply of data); role of funders for the systematic review. | 11 |

# Supplementary Tables

## Supplementary Table S1 Search Strategy

| Databases | Search items |
| --- | --- |
| Pubmed | (((((((((((((((((((((((((((((((((Diabetes Mellitus, Type 2[MeSH Terms]) OR (Diabetes Mellitus, Noninsulin-Dependent[Title/Abstract])) OR (Diabetes Mellitus, Ketosis-Resistant[Title/Abstract])) OR (Diabetes Mellitus, Ketosis Resistant[Title/Abstract])) OR (Ketosis-Resistant Diabetes Mellitus[Title/Abstract])) OR (Diabetes Mellitus, Non Insulin Dependent[Title/Abstract])) OR (Diabetes Mellitus, Non-Insulin-Dependent[Title/Abstract])) OR (Non-Insulin-Dependent Diabetes Mellitus[Title/Abstract])) OR (Diabetes Mellitus, Stable[Title/Abstract])) OR (Stable Diabetes Mellitus[Title/Abstract])) OR (Diabetes Mellitus, Type II[Title/Abstract])) OR (NIDDM[Title/Abstract])) OR (Diabetes Mellitus, Noninsulin Dependent[Title/Abstract])) OR (Diabetes Mellitus, Maturity-Onset[Title/Abstract])) OR (Diabetes Mellitus, Maturity Onset[Title/Abstract])) OR (Maturity-Onset Diabetes Mellitus[Title/Abstract])) OR (Maturity Onset Diabetes Mellitus[Title/Abstract])) OR (MODY[Title/Abstract])) OR (Diabetes Mellitus, Slow-Onset[Title/Abstract])) OR (Diabetes Mellitus, Slow Onset[Title/Abstract])) OR (Slow-Onset Diabetes Mellitus[Title/Abstract])) OR (Type 2 Diabetes Mellitus[Title/Abstract])) OR (Noninsulin-Dependent Diabetes Mellitus[Title/Abstract])) OR (Noninsulin Dependent Diabetes Mellitus[Title/Abstract])) OR (Maturity-Onset Diabetes[Title/Abstract])) OR (Diabetes, Maturity-Onset[Title/Abstract])) OR (Maturity Onset Diabetes[Title/Abstract])) OR (Type 2 Diabetes[Title/Abstract])) OR (Diabetes, Type 2[Title/Abstract])) OR (Diabetes Mellitus, Adult-Onset[Title/Abstract])) OR (Adult-Onset Diabetes Mellitus[Title/Abstract])) OR (Diabetes Mellitus, Adult Onset[Title/Abstract])) AND (((Hyperuricemia[MeSH Terms]) OR (Gout[MeSH Terms])) OR (Gouts[Title/Abstract]))) AND (((((((((((((((Medicine, Chinese Traditional[MeSH Terms]) OR (Traditional Chinese Medicine[Title/Abstract])) OR (Chung I Hsueh[Title/Abstract])) OR (Hsueh, Chung I[Title/Abstract])) OR (Traditional Medicine, Chinese[Title/Abstract])) OR (Zhong Yi Xue[Title/Abstract])) OR (Chinese Traditional Medicine[Title/Abstract])) OR (Chinese Medicine, Traditional[Title/Abstract])) OR (Traditional Tongue Diagnosis[Title/Abstract])) OR (Tongue Diagnoses, Traditional[Title/Abstract])) OR (Tongue Diagnosis, Traditional[Title/Abstract])) OR (Traditional Tongue Diagnoses[Title/Abstract])) OR (Traditional Tongue Assessment[Title/Abstract])) OR (Tongue Assessment, Traditional[Title/Abstract])) OR (Traditional Tongue Assessments[Title/Abstract])) |
| Cochrane Library | #1 MeSH descriptor: [Diabetes Mellitus, Type 2] explode all trees |
|  | #2 (Adult-Onset Diabetes Mellitus OR Maturity-Onset Diabetes OR Diabetes Mellitus, Noninsulin Dependent OR MODY OR Diabetes, Type 2 OR Diabetes Mellitus, Adult Onset OR Maturity Onset Diabetes OR Stable Diabetes Mellitus OR Diabetes Mellitus, Maturity Onset OR Diabetes Mellitus, Ketosis Resistant OR Diabetes Mellitus, Slow-Onset OR Diabetes Mellitus, Non Insulin Dependent OR Slow-Onset Diabetes Mellitus OR Diabetes Mellitus, Type II OR Maturity Onset Diabetes Mellitus OR Ketosis-Resistant Diabetes Mellitus OR Diabetes Mellitus, Adult-Onset OR Diabetes Mellitus, Stable OR Type 2 Diabetes Mellitus OR Diabetes Mellitus, Ketosis-Resistant OR Diabetes Mellitus, Non-Insulin-Dependent OR Noninsulin-Dependent Diabetes Mellitus OR Type 2 Diabetes OR Diabetes Mellitus, Noninsulin-Dependent OR Diabetes Mellitus, Slow Onset OR NIDDM OR Non-Insulin-Dependent Diabetes Mellitus OR Diabetes, Maturity-Onset OR Diabetes Mellitus, Maturity-Onset OR Noninsulin Dependent Diabetes Mellitus OR Maturity-Onset Diabetes Mellitus):ti,ab,kw (Word variations have been searched) |
|  | #3 #1 OR #2 |
|  | #4 MeSH descriptor: [Hyperuricemia] explode all trees |
|  | #5 MeSH descriptor: [Gout] explode all trees |
|  | #6 (Gouts):ti,ab,kw (Word variations have been searched) |
|  | #7 #4 OR #5 OR #6 |
|  | #8 MeSH descriptor: [Medicine, Chinese Traditional] explode all trees |
|  | #9 (Traditional Tongue Diagnosis OR Tongue Diagnoses, Traditional OR Traditional Tongue Assessments OR Tongue Diagnosis, Traditional OR Traditional Tongue Assessment OR Tongue Assessment, Traditional OR Traditional Tongue Diagnoses OR Hsueh, Chung I OR Traditional Medicine, Chinese OR Traditional Chinese Medicine OR Chinese Traditional Medicine OR Chinese Medicine, Traditional OR Chung I Hsueh OR Zhong Yi Xue):ti,ab,kw (Word variations have been searched) |
|  | #10 #8 OR #9 |
|  | #11 #3 AND #7 AND #10 |
| Embase | #1 'non insulin dependent diabetes mellitus'/exp |
|  | #2 ('adult onset diabetes':ab,ti OR 'adult onset diabetes mellitus':ab,ti OR 'diabetes mellitus type 2':ab,ti OR 'diabetes mellitus type ii':ab,ti OR 'diabetes mellitus, maturity onset':ab,ti OR 'diabetes mellitus, non insulin dependent':ab,ti OR 'diabetes mellitus, non-insulin-dependent':ab,ti OR 'diabetes mellitus, type 2':ab,ti OR 'diabetes mellitus, type ii':ab,ti OR 'diabetes type 2':ab,ti OR 'diabetes type ii':ab,ti OR 'diabetes, adult onset':ab,ti OR 'dm 2':ab,ti OR 'insulin independent diabetes':ab,ti OR 'insulin independent diabetes mellitus':ab,ti OR 'ketosis resistant diabetes mellitus':ab,ti OR 'maturity onset diabetes':ab,ti OR 'maturity onset diabetes mellitus':ab,ti OR 'maturity onset diabetes of the young':ab,ti OR niddm:ab,ti) AND 'non insulin dependent diabetes mellitus':ab,ti OR 'non insulin dependent diabetes':ab,ti OR 'non-insulin-dependent diabetes mellitus':ab,ti OR 'noninsulin dependent diabetes':ab,ti OR 'noninsulin dependent diabetes mellitus':ab,ti OR t2dm:ab,ti OR 'type 2 diabetes':ab,ti OR 'type 2 diabetes mellitus':ab,ti OR 'type ii diabetes':ab,ti OR 'type ii diabetes mellitus':ab,ti |
|  | #3 #1 OR #2 |
|  | #4 'hyperuricemia'/exp |
|  | #5 hyperuricacidaemia:ab,ti OR hyperuricacidemia:ab,ti OR hyperuricaemia:ab,ti OR 'senile hyperuricaemia':ab,ti OR 'senile hyperuricemia':ab,ti |
|  | #6 'gout'/exp |
|  | #7 arthragra:ab,ti OR 'arthritis urica':ab,ti OR 'arthritis, gouty':ab,ti OR cheiragra:ab,ti OR chiragra:ab,ti OR 'gouty arthritis':ab,ti OR 'gouty attack':ab,ti OR 'urate inflammation':ab,ti OR 'uric arthritis':ab,ti |
|  | #8 #4 OR #5 OR #6 OR #7 |
|  | #9 'chinese medicine'/exp |
|  | #10 'chinese herbal medicine':ab,ti OR 'chinese traditional medicine':ab,ti OR 'medicine, chinese traditional':ab,ti OR 'traditional chinese medicine':ab,ti |
|  | #11 #9 OR #10 |
|  | #12 #3 AND #8 AND #11 |
| Web of Science | #2 ((TS=(Hyperuricemia)) OR TS=(Gout)) OR TI=(Gouts) |
|  | #3 (((((((((((((((TS=(Medicine, Chinese Traditional)) OR ALL=(Traditional Chinese Medicine)) OR ALL=(Chung I Hsueh)) OR ALL=(Hsueh, Chung I)) OR ALL=(Traditional Medicine, Chinese)) OR ALL=(Zhong Yi Xue)) OR ALL=(Chinese Traditional Medicine)) OR ALL=(Chinese Medicine, Traditional)) OR ALL=(Traditional Tongue Diagnosis)) OR ALL=(Tongue Diagnoses, Traditional)) OR ALL=(Tongue Diagnosis, Traditional)) OR ALL=(Traditional Tongue Diagnoses)) OR ALL=(Traditional Tongue Diagnoses)) OR ALL=(Tongue Assessment, Traditional)) OR ALL=(Traditional Tongue Assessments)) |
|  | #4 #1 AND #2 AND #3 |
| CNKI | (SU='2型糖尿病'+'T2DM') AND (SU='高尿酸血症'+'痛风') AND (FT='中医'+'中药'+'中医药'+'中⻄医'+'中草药''+‘中成药’) |
| Wanfang Database | (主题:("2型糖尿病" or "T2DM") or 题名或关键词:("2型糖尿病" or "T2DM") or 摘要:("2型糖尿病" or "T2DM")) and (主题:("高尿酸血症" or "痛风") or 题名或关键词:("高尿酸血症" or"痛风") or 摘要:("高尿酸血症" or "痛风")) and (主题:("中医" or "中药" or "中医药" or "中⻄医"or "中草药" or "中成药")or 题名或关键词:("中医" or "中药" or "中医药" or "中⻄医" or "中草药" or "中成药") or 摘要:("中医" or "中药" or"中医药" or "中⻄医" or "中草药" or "中成药")) |
| CBM | ("2型糖尿病"[摘要:智能] OR "T2DM"[摘要:智能]) AND ("高尿酸血症"[摘要:智能] OR "痛风"[摘要:智能]) AND ("中医"[全部字段:智能] OR "中药"[全部字段:智能] OR "中医药"[全部字段:智能] OR "中⻄医"[全部字段:智能] OR "中草药"[全部字段:智能] OR "中成药"[全部字段:智能]) |
| VIP | ((M=2型糖尿病 OR T2DM) OR (K=2型糖尿病 OR T2DM) OR (R=2型糖尿病 OR T2DM)) AND ((M=高尿酸血症 OR 痛风) OR (K=高尿酸血症 OR 痛风) OR (R=高尿酸血症 OR 痛风)) AND ((M=中医 OR 中药 OR 中医药 OR 中⻄医 OR 中草药 OR 中成药) OR (K=中医 OR 中 药 OR 中医药 OR 中⻄医 OR 中草药 OR 中成药) OR (R=中医 OR 中药 OR 中医药 OR 中⻄医 OR 中草药 OR 中成药)) |

## Supplementary Table S2 Detailed components of CHM

| **study** | **chinese herb medicine** | **ingredients of herb prescriptin** | | **usage** |
| --- | --- | --- | --- | --- |
|  |  | **Latin name** | **Chinese name** |  |
| Gu et al. (2021) | Qingli Xiaotong formula | *Lonicera japonica* Thunb. [Caprifoliaceae; Lonice raejaponicae caulis], 20g *Alisma plantago-aquatica* L. [Alismataceae; Alismatis rhizoma], 20g *Platycodon grandiflorus* (Jacq.) A.DC. [Campanulaceae; Platycodonis radix], 20g *Clematis chinensis* Osbeck [Ranunculaceae; Clematidis radix et rhizoma], 20g *Astragalus mongholicus* Bunge [Fabaceae; Astragali radix], 20g *Dioscorea septemloba* Thunb. [Dioscoreaceae; Dioscoreae hypoglaucae rhizoma], 20g *Smilax glabra* Roxb. [Smilacaceae; Smilacis glabrae rhizoma], 20g *Carthamus tinctorius* L. [Asteraceae; Carthami flos], 20g *Pheretima aspergillum* (E.Perrier) [Pheretima], 20g *Rehmannia glutinosa* (Gaertn.) DC. [Orobanchaceae; Rehmanniae radix], 15g *Coix lacryma-jobi* L. [Poaceae; Coicis semen], 30g *Glycyrrhiza uralensis* Fisch. ex DC. [Fabaceae; Glycyrrhizae radix et rhizoma], 10g | Rendongteng, 20g Zexie, 20g Jiegeng, 20g Weilingxian, 20g Huangqi, 20g Bixie, 20g Tufuling, 20g Honghua, 20g Dilong, 20g Shengdihuang, 15g Chaoyiyiren, 30g Gancao, 10g | 75 ml bid |
| Hou (2019) | Kaiyu Yunpi decoction | *Bupleurum chinense* DC. [Apiaceae; Bupleuri radix], 12g *Curcuma aromatica* Salisb. [Zingiberaceae; Curcumae radix], 15g *Codonopsis pilosula* (Franch.) Nannf. [Campanulaceae; Codonopsis radix], 30g *Atractylodes macrocephala* Koidz. [Asteraceae; Atractylodis macrocephalae rhizoma], 15g *Coptis chinensis* Franch. [Ranunculaceae; Coptidis rhizoma], 12g *Nelumbo nucifera* Gaertn. [Nelumbonaceae; Nelumbinis folium], 15g *Senna tora* (L.) Roxb. [Fabaceae; Cassiae semen], 15g *Salvia miltiorrhiza* Bunge [Lamiaceae; Salviae miltiorrhizae radix et rhizoma], 20g *Pueraria montana* var. *lobata* (Willd.) Maesen & S.M.Almeida ex Sanjappa & Predeep [Fabaceae; Puerariae lobatae radix], 20g *Gleditsia sinensis* Lam. [Fabaceae; Gleditsiae spina], 9g *Glycyrrhiza uralensis* Fisch. ex DC. [Fabaceae; Glycyrrhizae radix et rhizoma], 6g | Chaihu, 12g Yujin, 15g Dangshen, 30g Baizhu, 15g Huanglian, 12g Heye, 15g Juemingzi, 15g Danshen, 20g Gegen, 20g Zaojiaoci, 9g Gancao, 6g | 100 ml bid |
| Hu et al. (2019) | Self−made Jianpi Yishen Huazhuo decoction | *Astragalus mongholicus* Bunge [Fabaceae; Astragali radix], 20g *Salvia miltiorrhiza* Bunge [Lamiaceae; Salviae miltiorrhizae radix et rhizoma], 15g *Rehmannia glutinosa* (Gaertn.) DC. [Orobanchaceae; Rehmanniae radix praeparata], 15g *Codonopsis pilosula* (Franch.) Nannf. [Campanulaceae; Codonopsis radix], 20g *Atractylodes macrocephala* Koidz. [Asteraceae; Atractylodis macrocephalae rhizoma], 10g *Wolfiporia cocos* (F.A. Wolf) Ryvarden & Gilb., 10g *Rheum palmatum* L. [Polygonaceae; Rhei radix et rhizoma], 10g *Neolitsea cassia* (L.) Kosterm. [Lauraceae; Cinnamomi ramulus], 10g *Cornus officinalis* Siebold & Zucc. [Cornaceae; Corni fructus], 15g *Dioscorea septemloba* Thunb. [Dioscoreaceae; Dioscoreae hypoglaucae rhizoma], 10g *Alisma plantago-aquatica* L. [Alismataceae; Alismatis rhizoma], 10g *Prunus persica* (L.) Batsch [Rosaceae; Persicae semen], 10g *Carthamus tinctorius* L. [Asteraceae; Carthami flos], 10g | Huangqi, 20g Danshen, 15g Shudihuang, 15g Dangshen, 20g Baizhu, 10g Fuling, 10g Shudahuang, 10g Guizhi, 10g Shanzhuyu, 15g Bixie, 10g Zexie, 10g Taoren, 10g Honghua, 10g | 100 ml bid |
| Liu (2020) | Qiwei Baizhu powder | *Codonopsis pilosula* (Franch.) Nannf. [Campanulaceae; Codonopsis radix], 15g *Atractylodes macrocephala* Koidz. [Asteraceae; Atractylodis macrocephalae rhizoma], 15g *Wolfiporia cocos* (F.A. Wolf) Ryvarden & Gilb., 30g *Glycyrrhiza uralensis* Fisch. ex DC. [Fabaceae; Glycyrrhizae radix et rhizoma], 10g *Pogostemon cablin* (Blanco) Benth. [Lamiaceae; Pogostemonis herba], 10g *Dolomiaea costus* (Falc.) Kasana & A.K.Pandey [Asteraceae; Aucklandiae radix], 10g *Pueraria montana* var. *lobata* (Willd.) Maesen & S.M.Almeida ex Sanjappa & Predeep [Fabaceae; Puerariae lobatae radix], 30g | Dangshen, 15g Baizhu, 15g Fuling, 30g Gancao, 10g Huoxiang, 10g Muxiang, 10g Gegen, 30g | 0.5 package bid |
| Liu (2021) | Jianpi Yishen Huazhuo decoction | *Astragalus mongholicus* Bunge [Fabaceae; Astragali radix], 20g *Salvia miltiorrhiza* Bunge [Lamiaceae; Salviae miltiorrhizae radix et rhizoma], 12g *Cornus officinalis* Siebold & Zucc. [Cornaceae; Corni fructus], 12g *Rehmannia glutinosa* (Gaertn.) DC. [Orobanchaceae; Rehmanniae radix praeparata], 9g *Codonopsis pilosula* (Franch.) Nannf. [Campanulaceae; Codonopsis radix], 15g *Atractylodes macrocephala* Koidz. [Asteraceae; Atractylodis macrocephalae rhizoma], 9g *Rheum palmatum* L. [Polygonaceae; Rhei radix et rhizoma], 9g *Neolitsea cassia* (L.) Kosterm. [Lauraceae; Cinnamomi ramulus], 12g *Wolfiporia cocos* (F.A. Wolf) Ryvarden & Gilb., 12g *Dioscorea septemloba* Thunb. [Dioscoreaceae; Dioscoreae hypoglaucae rhizoma], 5g *Alisma plantago-aquatica* L. [Alismataceae; Alismatis rhizoma], 9g *Prunus persica* (L.) Batsch [Rosaceae; Persicae semen], 5g *Carthamus tinctorius* L. [Asteraceae; Carthami flos], 10g | Huangqi, 20g Danshen, 12g Shanzhuyu, 12g Shudihuang, 9g Dangshen, 15g Baizhu, 9g Shudahuang, 9g Guizhi, 12g Fuling, 12g Bixie, 5g Zexie, 9g Taoren, 5g Honghua, 10g | 175 ml bid |
| Lou (2017) | Modified Baihu Erdi decoction | Gypsum fibrosum, 20g *Anemarrhena asphodeloides* Bunge [Asparagaceae; Anemarrhenae rhizoma], 10g *Rehmannia glutinosa* (Gaertn.) DC. [Orobanchaceae; Rehmanniae radix], 15g *Euphorbia humifusa* Willd. [Euphorbiaceae; Euphorbiae humifusae herba], 10g *Lycium chinense* Mill. [Solanaceae; Lycii cortex], 10g *Coptis chinensis* Franch. [Ranunculaceae; Coptidis rhizoma], 6g *Euonymus alatus* (Thunb.) Siebold [Celastraceae; Ramulus euonymi], 10g *Morus alba* L. [Moraceae; Mori folium], 6g *Phellodendron chinense* C.K.Schneid. [Rutaceae; Phellodendri chinensis cortex], 10g *Coix lacryma-jobi* L. [Poaceae; Coicis semen], 20g *Alisma plantago-aquatica* L. [Alismataceae; Alismatis rhizoma], 10g *Achyranthes bidentata* Blume [Amaranthaceae; Achyranthis bidentatae radix], 10g *Atractylodes lancea* (Thunb.) DC. [Asteraceae; Atractylodis rhizoma], 10g *Salvia miltiorrhiza* Bunge [Lamiaceae; Salviae miltiorrhizae radix et rhizoma], 10g *Glycyrrhiza uralensis* Fisch. ex DC. [Fabaceae; Glycyrrhizae radix et rhizoma praeparata cum melle], 6g | Shengshigao, 20g Zhimu, 10g Shengdihuang, 15g Dijincao, 10g Digupi, 10g Chuanlian, 6g Guijianyu, 10g Sangye, 6g Huangbo, 10g Shengyiren, 20g Zexie, 10g Niuxi, 10g Cangzhu, 10g Danshen, 10g Zhigancao, 6g | 200 ml bid |
| Ma et al. (2020) | Qingli Xiaotong formula | *Lonicera japonica* Thunb. [Caprifoliaceae; Lonice raejaponicae caulis], 20g *Rehmannia glutinosa* (Gaertn.) DC. [Orobanchaceae; Rehmanniae radix], 15g *Coix lacryma-jobi* L. [Poaceae; Coicis semen], 30g *Alisma plantago-aquatica* L. [Alismataceae; Alismatis rhizoma], 20g *Platycodon grandiflorus* (Jacq.) A.DC. [Campanulaceae; Platycodonis radix], 20g *Clematis chinensis* Osbeck [Ranunculaceae; Clematidis radix et rhizoma], 20g *Astragalus mongholicus* Bunge [Fabaceae; Astragali radix], 20g *Dioscorea septemloba* Thunb. [Dioscoreaceae; Dioscoreae hypoglaucae rhizoma], 20g *Smilax glabra* Roxb. [Smilacaceae; Smilacis glabrae rhizoma], 20g *Carthamus tinctorius* L. [Asteraceae; Carthami flos], 20g *Pheretima aspergillum* (E.Perrier) [Pheretima], 20g *Glycyrrhiza uralensis* Fisch. ex DC. [Fabaceae; Glycyrrhizae radix et rhizoma], 10g | Rendongteng, 20g Shengdihuang, 15g Chaoyiyiren, 30g Zexie, 20g Jiegeng, 20g Weilingxian, 20g Huangqi, 20g Bixie, 20g Tufuling, 20g Honghua, 20g Dilong, 20g Gancao, 10g | 150 ml bid |
| Peng et al.  (2014) | Modified Dachaihu decoction | *Bupleurum chinense* DC. [Apiaceae; Bupleuri radix] *Scutellaria baicalensis* Georgi [Lamiaceae; Scutellariae radix] *Pinellia ternata* (Thunb.) Makino [Araceae; Pinelliae rhizoma] *Paeonia lactiflora* Pall. [Paeoniaceae; Paeoniae radix alba] *Rheum palmatum* L. [Polygonaceae; Rhei radix et rhizoma] *Citrus × aurantium* L. [Rutaceae; Aurantii fructus immaturus] *Crataegus pinnatifida* Bunge [Rosaceae; Crataegi fructus] *Monascus purpureus* Went. *Zingiber officinale* Roscoe [Zingiberaceae; Zingiberis rhizoma] *Coptis chinensis* Franch. [Ranunculaceae; Coptidis rhizoma] | Chaihu Huangqin Banxia Baishaoyao Dahuang Zhishi Shengshanzha Hongqu Ganjiang Huanglian | 0.5 package bid |
| Shao (2020) | Jiedu Tongluo Baoshen Jiangzhuo formula | *Coptis chinensis* Franch. [Ranunculaceae; Coptidis rhizoma], 20g *Phellodendron chinense* C.K.Schneid. [Rutaceae; Phellodendri chinensis cortex], 15g *Smilax glabra* Roxb. [Smilacaceae; Smilacis glabrae rhizoma], 25g *Dioscorea septemloba* Thunb. [Dioscoreaceae; Dioscoreae hypoglaucae rhizoma], 20g *Salvia miltiorrhiza* Bunge [Lamiaceae; Salviae miltiorrhizae radix et rhizoma], 15g *Rheum palmatum* L. [Polygonaceae; Rhei radix et rhizoma], 6g *Cornus officinalis* Siebold & Zucc. [Cornaceae; Corni fructus], 15g *Coix lacryma-jobi* L. [Poaceae; Coicis semen], 20g *Atractylodes lancea* (Thunb.) DC. [Asteraceae; Atractylodis rhizoma], 10g *Fraxinus chinensis* subsp. *rhynchophylla* (Hance) A.E.Murray [Oleaceae; Fraxini cortex], 10g *Clematis chinensis* Osbeck [Ranunculaceae; Clematidis radix et rhizoma], 10g *Achyranthes bidentata* Blume [Amaranthaceae; Achyranthis bidentatae radix], 10g *Glycyrrhiza uralensis* Fisch. ex DC. [Fabaceae; Glycyrrhizae radix et rhizoma], 10g | Huanglian, 20g Huangbo, 15g Tufuling, 25g Bixie, 20g Danshen, 15g Jiudahuang, 6g Shanyurou, 15g Yiyiren, 20g Cangzhu, 10g Qinpi, 10g Weilingxian, 10g Niuxi, 10g Gancao, 10g | 150 ml bid |
| Tang (2014) | Huazhuo decoction | *Astragalus mongholicus* Bunge [Fabaceae; Astragali radix], 30g *Codonopsis pilosula* (Franch.) Nannf. [Campanulaceae; Codonopsis radix], 12g *Atractylodes macrocephala* Koidz. [Asteraceae; Atractylodis macrocephalae rhizoma], 15g *Wolfiporia cocos* (F.A. Wolf) Ryvarden & Gilb., 15g *Rehmannia glutinosa* (Gaertn.) DC. [Orobanchaceae; Rehmanniae radix], 12g *Cornus officinalis* Siebold & Zucc. [Cornaceae; Corni fructus], 30g *Lycium barbarum* L. [Solanaceae; Lycii fructus], 21g *Epimedium brevicornu* Maxim. [Berberidaceae; Epimedii folium], 30g *Alisma plantago-aquatica* L. [Alismataceae; Alismatis rhizoma], 12g *Atractylodes lancea* (Thunb.) DC. [Asteraceae; Atractylodis rhizoma], 9g *Smilax glabra* Roxb. [Smilacaceae; Smilacis glabrae rhizoma], 15g *Plantago asiatica* L. [Plantaginaceae; Plantaginis semen], 12g *Rheum palmatum* L. [Polygonaceae; Rhei radix et rhizoma], 3g *Prunus persica* (L.) Batsch [Rosaceae; Persicae semen], 9g *Carthamus tinctorius* L. [Asteraceae; Carthami flos], 9g | Huangqi, 30g Dangshen, 12g Baizhu, 15g Fuling, 15g Shengdi, 12g Shanyurou, 30g Gouqizi, 21g Yinyanghuo, 30g Zexie, 12g Cangzhu, 9g Tufuling, 15g Cheqianzi, 12g Jiudahuang, 3g Taoren, 9g Honghua, 9g | 200 ml bid |
| Tang et al.  (2017) | Yinshandan formula | *Lonicera japonica* Thunb. [Caprifoliaceae; Lonicerae japonicae flos], 10g *Salvia miltiorrhiza* Bunge [Lamiaceae; Salviae miltiorrhizae radix et rhizoma], 30g *Alisma plantago-aquatica* L. [Alismataceae; Alismatis rhizoma], 30g *Cremastra appendiculata* (D.Don) Makino [Orchidaceae; Cremastrae pseudobulbus pleiones pseudobulbus], 30g *Angelica sinensis* (Oliv.) Diels [Apiaceae; Angelicae sinensis radix], 10g *Lysimachia christinae* Hance [Primulaceae; Lysimachiae herba], 30g *Reynoutria japonica* Houtt. [Polygonaceae; Polygoni cuspidati rhizoma et radix], 15g *Aralia chinensis* L. [Araliaceae; Cortex erythrinae], 15g *Rheum palmatum* L. [Polygonaceae; Rhei radix et rhizoma], 10g *Boswellia sacra* Flück. [Burseraceae; Olibanum], 8g *Commiphora myrrha* (T.Nees) Engl. [Burseraceae; Myrrha], 8g | Jinyinhua, 10g Danshen, 30g Zexie, 30g Shancigu, 30g Danggui, 10g Jinqiancao, 30g Huzhang, 15g Haitongpi, 15g Dahuang, 10g Zhiruxiang, 8g Zhimoyao, 8g | 200 ml bid |
| Wu (2015) | Self−made Huanglian Zexie decoction | *Coix lacryma-jobi* L. [Poaceae; Coicis semen], 30g *Spatholobus suberectus* Dunn [Fabaceae; Spatholobi caulis], 30g *Smilax glabra* Roxb. [Smilacaceae; Smilacis glabrae rhizoma], 30g *Alisma plantago-aquatica* L. [Alismataceae; Alismatis rhizoma], 20g *Acorus calamus* var. *angustatus* Besser [Acoraceae; Acori tatarinowii rhizoma], 20g *Atractylodes macrocephala* Koidz. [Asteraceae; Atractylodis macrocephalae rhizoma], 20g *Dioscorea septemloba* Thunb. [Dioscoreaceae; Dioscoreae hypoglaucae rhizoma], 20g *Pheretima aspergillum* (E.Perrier) [Pheretima], 20g *Clematis chinensis* Osbeck [Ranunculaceae; Clematidis radix et rhizoma], 20g *Commiphora myrrha* (T.Nees) Engl. [Burseraceae; Myrrha], 15g *Cyathula officinalis* K.C.Kuan [Amaranthaceae; Cyathulae radix], 15g *Codonopsis pilosula* (Franch.) Nannf. [Campanulaceae; Codonopsis radix], 15g *Astragalus mongholicus* Bunge [Fabaceae; Astragali radix], 15 *Rheum palmatum* L. [Polygonaceae; Rhei radix et rhizoma], 10g *Hiptage benghalensis* (L.) Kurz [Malpighiaceae], 10g *Glycyrrhiza uralensis* Fisch. ex DC. [Fabaceae; Glycyrrhizae radix et rhizoma], 10g | Yiyiren, 30g Jixueteng, 30g Tufuling, 30g Zexie, 20g Shichangpu, 20g Baizhu, 20g Bixie, 20g Dilong, 20g Weilingxian, 20g Moyao, 15g Chuanniuxi, 15g Dangshen, 15g Huangqi, 15g Jiudahuang, 10g Honglong, 10g Shenggancao, 10g | 0.5 package bid |
| Xie et al.  (2021) | Lingbi Simiao decoction | *Smilax glabra* Roxb. [Smilacaceae; Smilacis glabrae rhizoma], 30g *Dioscorea septemloba* Thunb. [Dioscoreaceae; Dioscoreae hypoglaucae rhizoma], 15g *Atractylodes lancea* (Thunb.) DC. [Asteraceae; Atractylodis rhizoma], 15g *Phellodendron chinense* C.K.Schneid. [Rutaceae; Phellodendri chinensis cortex], 6g *Coix lacryma-jobi* L. [Poaceae; Coicis semen], 15g *Achyranthes bidentata* Blume [Amaranthaceae; Achyranthis bidentatae radix], 15g *Crataegus pinnatifida* Bunge [Rosaceae; Crataegi fructus] 15g *Atractylodes macrocephala* Koidz. [Asteraceae; Atractylodis macrocephalae rhizoma], 15g *Rehmannia glutinosa* (Gaertn.) DC. [Orobanchaceae; Rehmanniae radix praeparata], 15g *Bombyx mori* Linnaeus [Bombyx batryticatus], 9g *Neolitsea cassia* (L.) Kosterm. [Lauraceae; Cinnamomi ramulus], 9g *Plantago asiatica* L. [Plantaginaceae; Plantaginis herba], 15g | Tufuling, 30g Bixie, 15g Cangzhu, 15g Huangbo, 6g Yiyiren, 15g Huainiuxi, 15g Jiaoshanzha, 15g Baizhu, 15g Shudihuang, 15g Jiangcan, 9g Guizhi, 9g Cheqiancao, 15g | 150 ml bid |
| Yin (2015） | Huazhuo decoction | *Astragalus mongholicus* Bunge [Fabaceae; Astragali radix], 30g *Dioscorea oppositifolia* L. [Dioscoreaceae; Dioscoreae rhizoma], 15g *Smilax glabra* Roxb. [Smilacaceae; Smilacis glabrae rhizoma], 30g *Dioscorea septemloba* Thunb. [Dioscoreaceae; Dioscoreae hypoglaucae rhizoma], 30g *Plantago asiatica* L. [Plantaginaceae; Plantaginis herba], 15g *Reynoutria japonica* Houtt. [Polygonaceae; Polygoni cuspidati rhizoma et radix], 15g *Atractylodes lancea* (Thunb.) DC. [Asteraceae; Atractylodis rhizoma], 12g *Senna tora* (L.) Roxb. [Fabaceae; Cassiae semen], 15g *Rheum palmatum* L. [Polygonaceae; Rhei radix et rhizoma], 3g *Rehmannia glutinosa* (Gaertn.) DC. [Orobanchaceae; Rehmanniae radix], 12g *Reynoutria multiflora* (Thunb.) Moldenke [Polygonaceae; Polygoni multiflori radix], 12g *Pheretima aspergillum* (E.Perrier) [Pheretima], 9g *Prunus persica* (L.) Batsch [Rosaceae; Persicae semen], 9g *Zingiber officinale* Roscoe [Zingiberaceae; Zingiberis rhizoma], 6g | Shenghuangqi, 30g Chaoshanyao, 15g Tufuling, 30g Bixie, 30g Cheqiancao, 15g Huzhang, 15g Cangzhu, 12g Juemingzi, 15g Shudahuang, 3g Shengdi, 12g Heshouwu, 12g Dilong, 9g Taoren, 9g Ganjiang, 6g | 200 ml bid |
| Yu（2010) | Jianpi Huayu decoction | *Astragalus mongholicus* Bunge [Fabaceae; Astragali radix], 50g *Rehmannia glutinosa* (Gaertn.) DC. [Orobanchaceae; Rehmanniae radix], 30g *Salvia miltiorrhiza* Bunge [Lamiaceae; Salviae miltiorrhizae radix et rhizoma], 30g *Coix lacryma-jobi* L. [Poaceae; Coicis semen], 30g *Angelica sinensis* (Oliv.) Diels [Apiaceae; Angelicae sinensis radix], 15g *Reynoutria multiflora* (Thunb.) Moldenke [Polygonaceae; Polygoni multiflori radix], 15g *Epimedium brevicornu* Maxim. [Berberidaceae; Epimedii folium], 15g *Achyranthes bidentata* Blume [Amaranthaceae; Achyranthis bidentatae radix], 10g *Dioscorea septemloba* Thunb. [Dioscoreaceae; Dioscoreae hypoglaucae rhizoma], 10g *Pueraria montana* var. *lobata* (Willd.) Maesen & S.M.Almeida ex Sanjappa & Predeep [Fabaceae; Puerariae lobatae radix], 10g | Shenghuangqi, 50g Shengdi, 30g Danshen, 30g Yiren, 30g Danggui, 15g Heshouwu, 15g Xianlingpi, 15g Niuxi, 10g Bixie, 10g Gegen, 10g | 0.5 package bid |
| Zhang et al.  (2021) | Modified Dachaihu decoction | *Bupleurum chinense* DC. [Apiaceae; Bupleuri radix], 10g *Scutellaria baicalensis* Georgi [Lamiaceae; Scutellariae radix], 10g *Citrus × aurantium* L. [Rutaceae; Aurantii fructus immaturus], 10g *Paeonia lactiflora* Pall. [Paeoniaceae; Paeoniae radix alba], 20g *Atractylodes lancea* (Thunb.) DC. [Asteraceae; Atractylodis rhizoma], 15g *Phellodendron chinense* C.K.Schneid. [Rutaceae; Phellodendri chinensis cortex], 15g *Pinellia ternata* (Thunb.) Makino [Araceae; Pinelliae rhizoma praeparatum], 9g *Coix lacryma-jobi* L. [Poaceae; Coicis semen], 30g *Smilax glabra* Roxb. [Smilacaceae; Smilacis glabrae rhizoma], 30g *Bombyx mori* Linnaeus, 30g *Cremastra appendiculata* (D.Don) Makino [Orchidaceae; Cremastrae pseudobulbus pleiones pseudobulbus], 10g *Cyathula officinalis* K.C.Kuan [Amaranthaceae; Cyathulae radix], 15g | Chaihu, 10g Huangqin, 10g Zhishi, 10g Baishao, 20g Cangzhu, 15g Huangbo, 15g Fabanxia, 9g Shengyiyiren, 30g Tufuling, 30g Cansha, 30g Shancigu, 10g Chuanniuxi, 15g | 0.5 package bid |
| Zhou (2018) | Shenling Erzhu decoction | *Codonopsis pilosula* (Franch.) Nannf. [Campanulaceae; Codonopsis radix] *Wolfiporia cocos* (F.A. Wolf) Ryvarden & Gilb. *Atractylodes macrocephala* Koidz. [Asteraceae; Atractylodis macrocephalae rhizoma] *Atractylodes lancea* (Thunb.) DC. [Asteraceae; Atractylodis rhizoma] *Coix lacryma-jobi* L. [Poaceae; Coicis semen] *Achyranthes bidentata* Blume [Amaranthaceae; Achyranthis bidentatae radix] *Pinellia ternata* (Thunb.) Makino [Araceae; Pinelliae rhizoma praeparatum] *Citrus × aurantium* L. [Rutaceae; Citri reticulatae pericarpium] *Alisma plantago-aquatica* L. [Alismataceae; Alismatis rhizoma] *Dioscorea septemloba* Thunb. [Dioscoreaceae; Dioscoreae hypoglaucae rhizoma] *Lonicera japonica* Thunb. [Caprifoliaceae; Lonice raejaponicae caulis] *Nelumbo nucifera* Gaertn. [Nelumbonaceae; Nelumbinis folium] | Dangshen Fuling Baizhu Cangzhu Yiyiren Niuxi Fabanxia Chenpi Zexie Fenbixie Rendongteng Heye | 100 ml bid |
| Zou (2021) | Jianpi Yishen Huazhuo decoction | *Rheum palmatum* L. [Polygonaceae; Rhei radix et rhizoma], 3g *Zingiber officinale* Roscoe [Zingiberaceae; Zingiberis rhizoma], 6g *Pheretima aspergillum* (E.Perrier) [Pheretima], 9g *Prunus persica* (L.) Batsch [Rosaceae; Persicae semen], 9g *Atractylodes lancea* (Thunb.) DC. [Asteraceae; Atractylodis rhizoma], 12g *Rehmannia glutinosa* (Gaertn.) DC. [Orobanchaceae; Rehmanniae radix], 12g *Reynoutria multiflora* (Thunb.) Moldenke [Polygonaceae; Polygoni multiflori radix], 12g *Plantago asiatica* L. [Plantaginaceae; Plantaginis herba], 15g *Reynoutria japonica* Houtt. [Polygonaceae; Polygoni cuspidati rhizoma et radix], 15g *Senna tora* (L.) Roxb. [Fabaceae; Cassiae semen], 15g *Dioscorea oppositifolia* L. [Dioscoreaceae; Dioscoreae rhizoma], 15g *Smilax glabra* Roxb. [Smilacaceae; Smilacis glabrae rhizoma], 30g *Dioscorea septemloba* Thunb. [Dioscoreaceae; Dioscoreae hypoglaucae rhizoma], 30g *Astragalus mongholicus* Bunge [Fabaceae; Astragali radix], 30g | Shudahuang, 3g Ganjiang, 6g Dilong, 9g Taoren, 9g Cangzhu, 12g Shengdi, 12g Heshouwu, 12g Cheqiancao, 15g Huzhang, 15g Juemingzi, 15g Chaoshanyao, 15g Tufuling, 30g Bixie, 30g Shenghuangqi, 30g | 250 ml bid |

# Supplementary Figures

## Supplementary Figure 1. Forest plot for subgroup analysis.

## Supplementary Figure 1.1 Subgroup analysis for FBG and 2hPG.


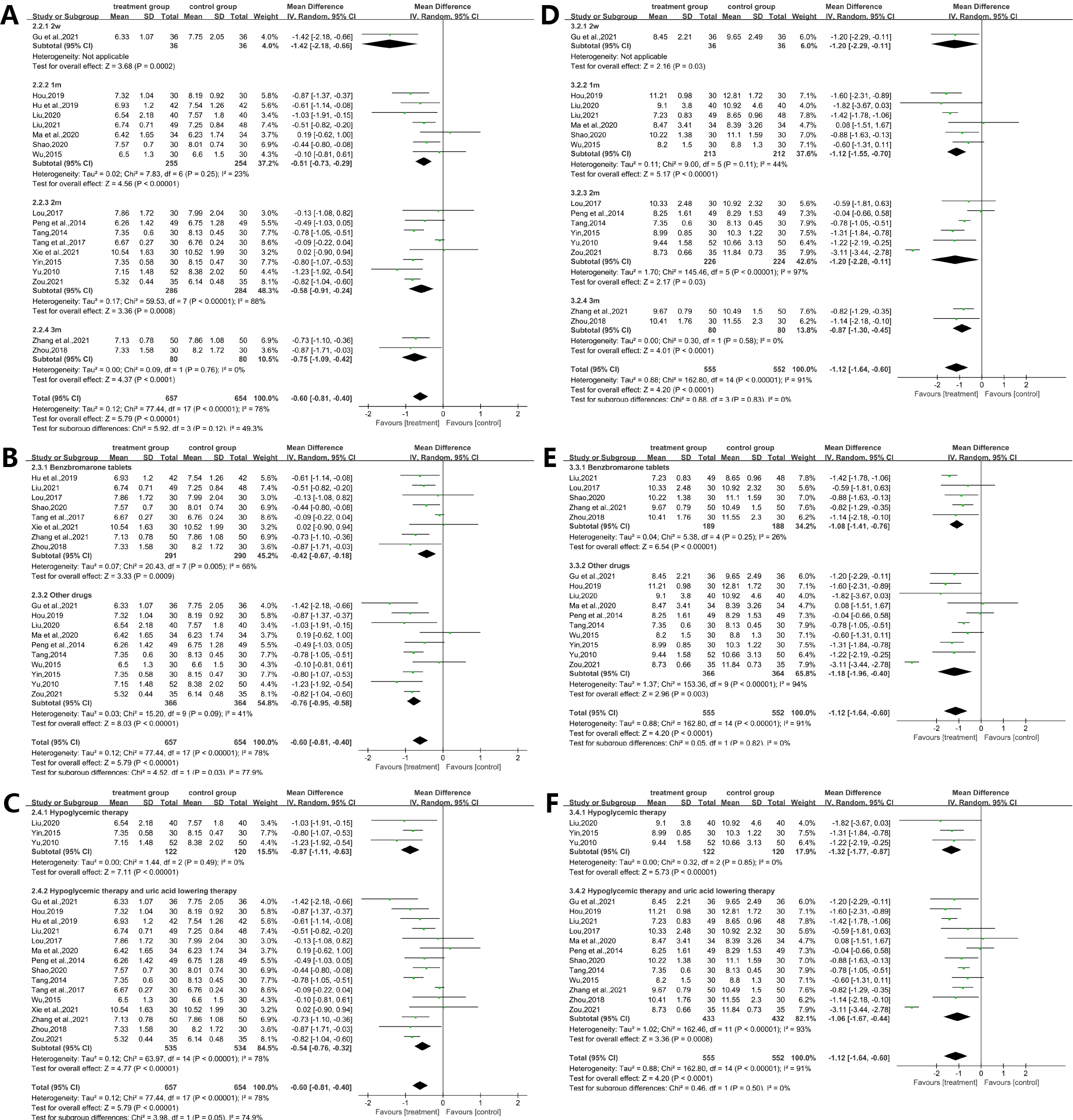


(A)-(C): subgroup analysis for FBG. (D)-(F): subgroup analysis for 2hPG.

## Supplementary Figure 1.2 Subgroup analysis for HbA1c and UA.


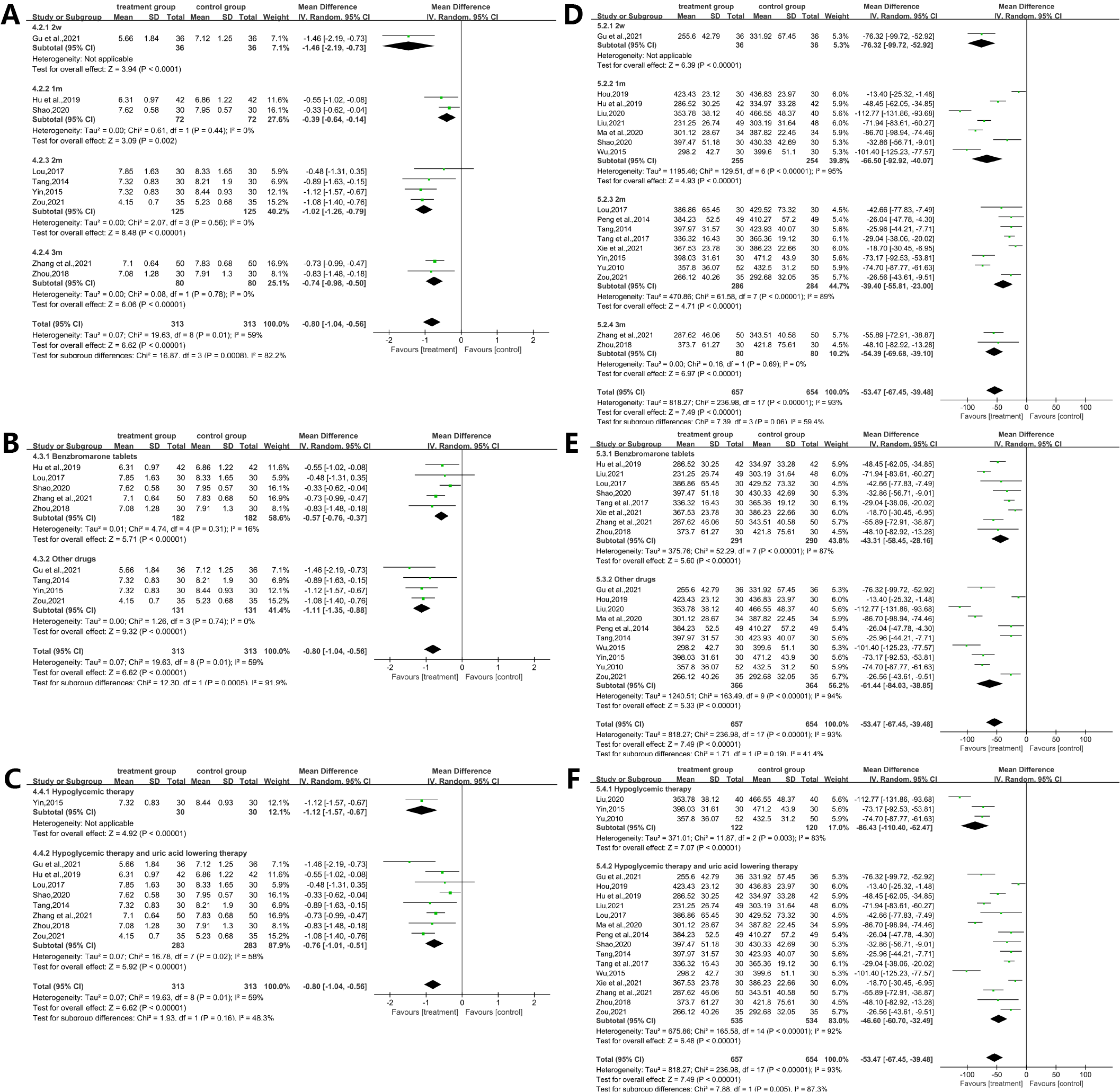


(A)-(C): subgroup analysis for HbA1c. (D)-(F): subgroup analysis for UA.

## Supplementary Figure 1.3 Subgroup analysis for TG and TC.


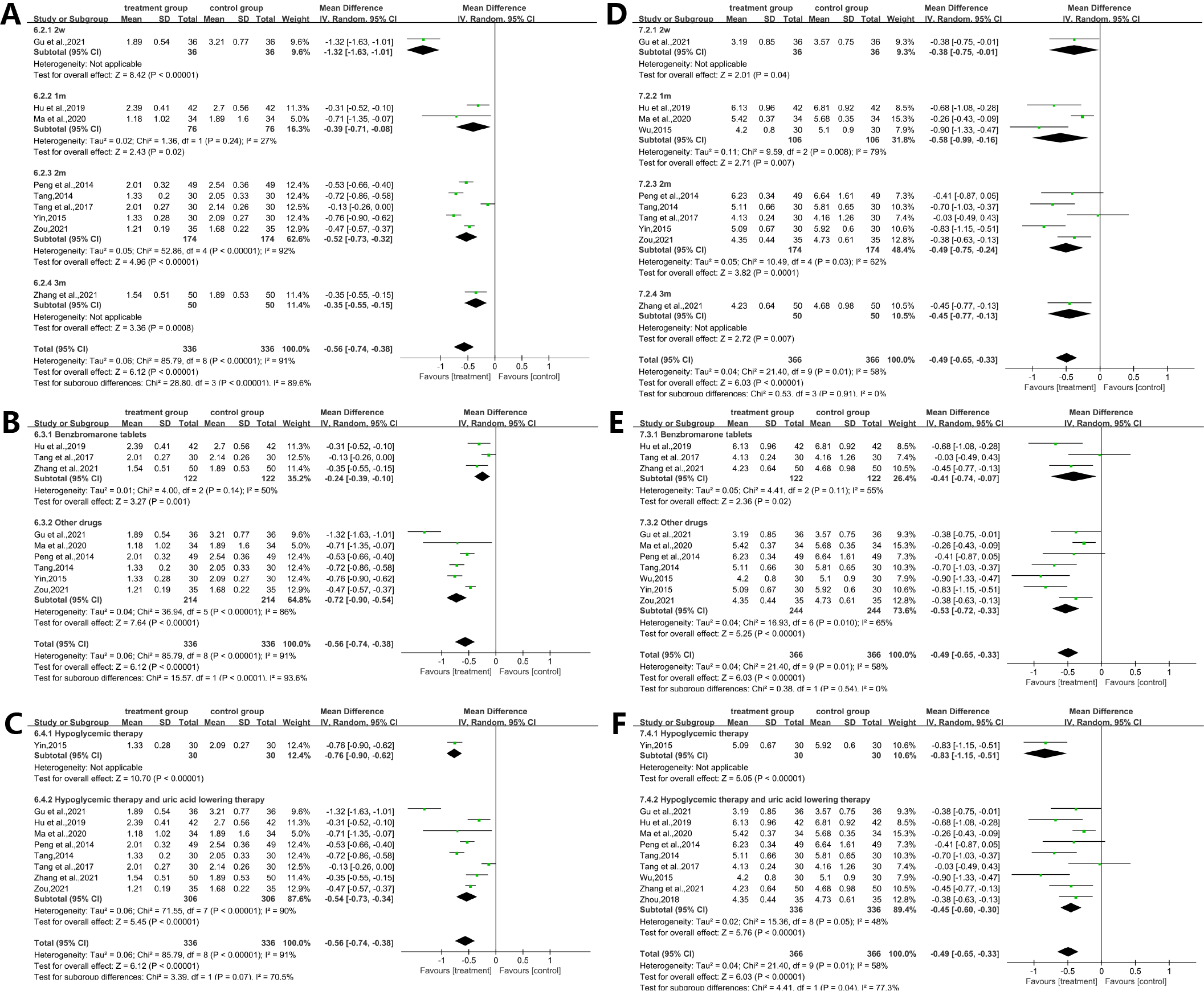


(A)-(C): subgroup analysis for TG. (D)-(F): subgroup analysis for TC.

## Supplementary Figure 1.4 Subgroup analysis for overall effective rate.


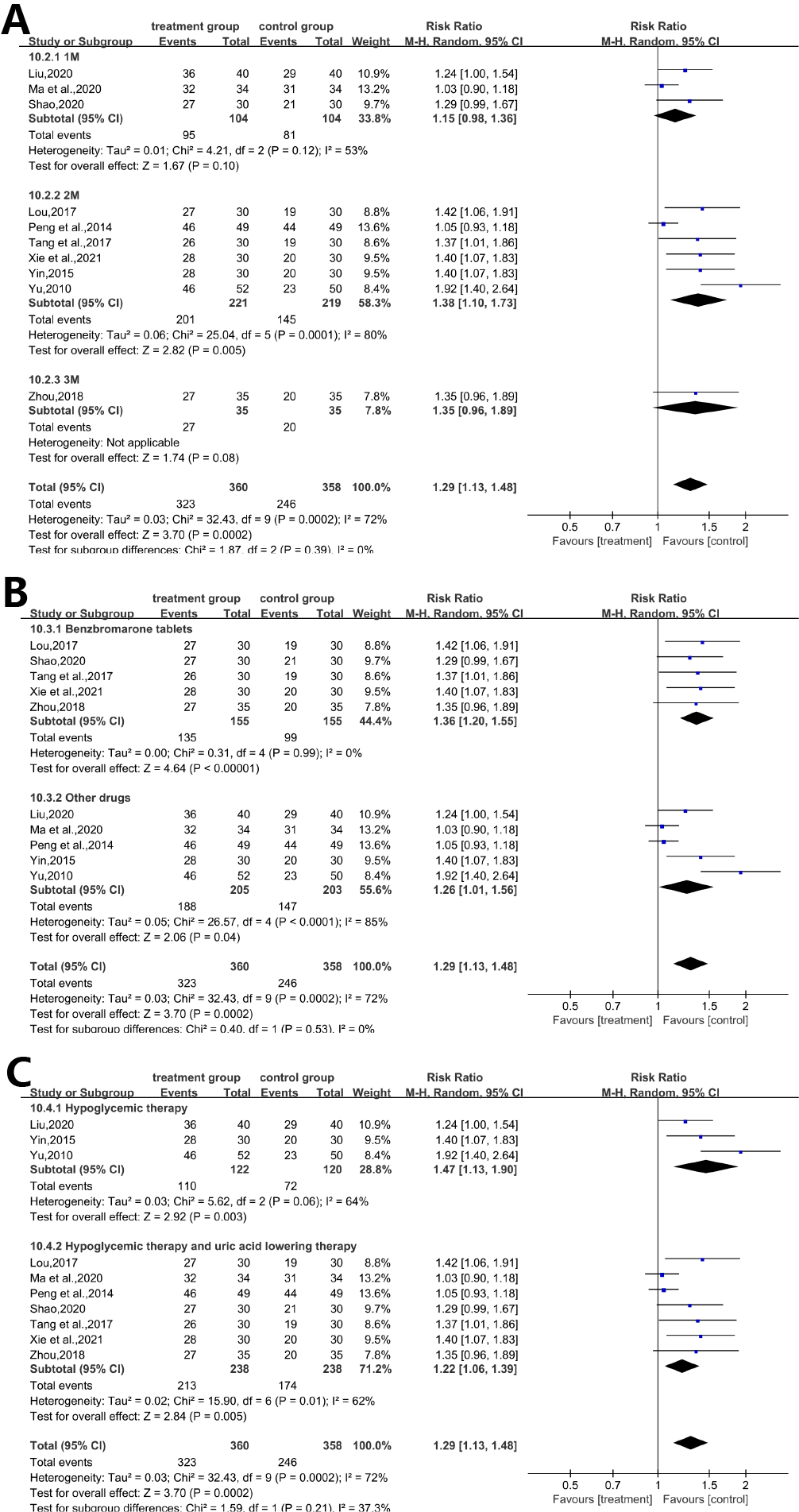


(A)-(C): subgroup analysis for overall effective rate.

## Supplementary Figure 2. Egger's test for assessing publication bias.


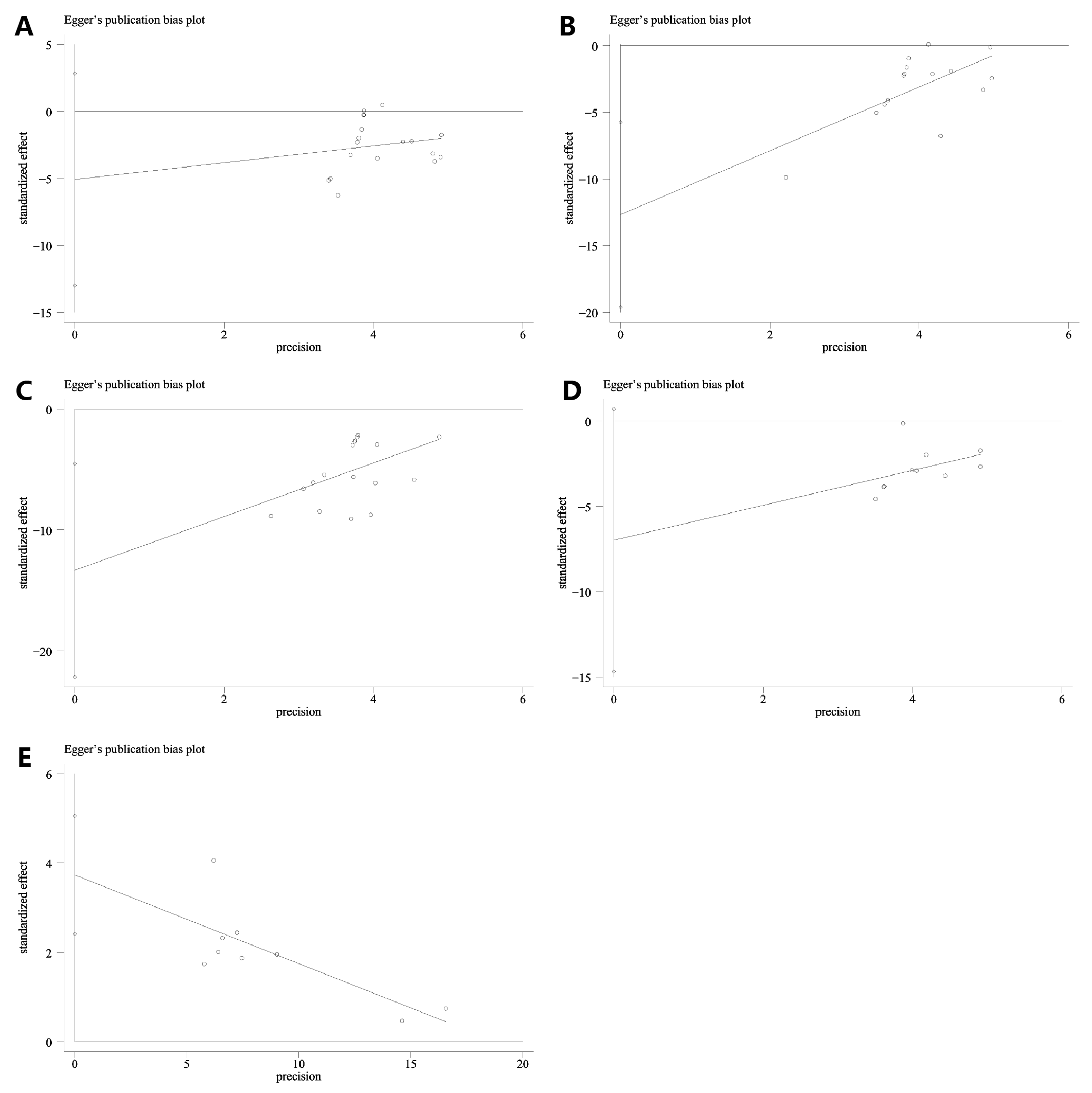


(A) FBG; (B) 2hPG; (C)UA; (D)TC; and (E) overall effective rate.

## Supplementary Figure 3. Sensitivity analysis.


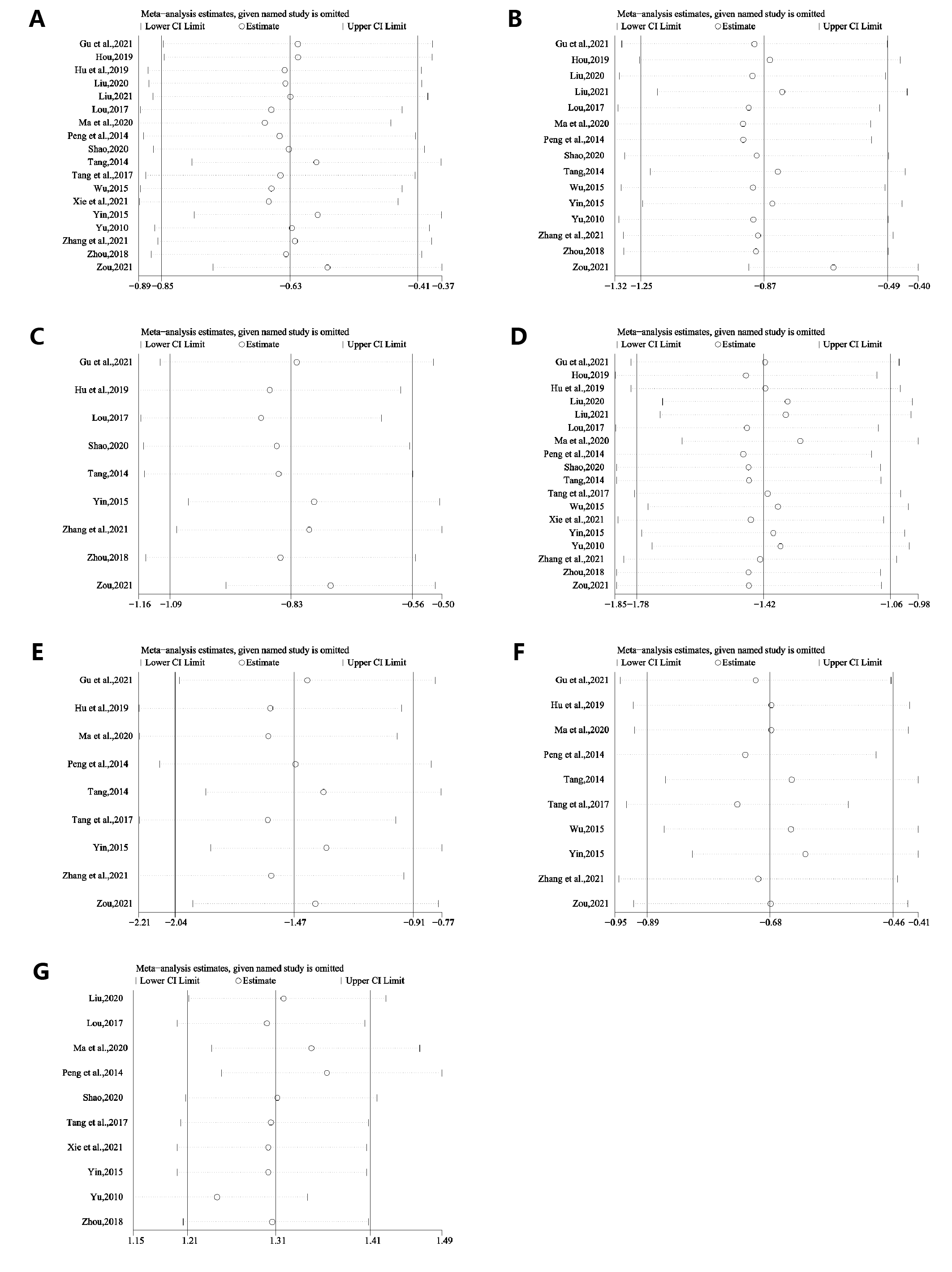


(A) FBG; (B) 2hPG; (C) HbA1c; (D) UA; (E) TG; (F) TC; and (G) overall effective rate.
